# A blockchain-based secure data transmission framework in IoT using adaptive deep network with optimized cryptography mechanism

**DOI:** 10.1038/s41598-026-50548-5

**Published:** 2026-05-11

**Authors:** Anguraju Krishnan, Rajesh Arunachalam, M. P. Rajakumar, A. Sahaya Anselin Nisha, Sumanth Venugopal, J. Yogapriya

**Affiliations:** 1 Department of Computer Science and Engineering, Saveetha School of Engineering, Saveetha Institute of Medical and Technical Sciences, Thandalam, Chennai, 602105 Tamil Nadu India; 2 Department of Electronics and Communication Engineering, Saveetha School of Engineering, Saveetha Institute of Medical and Technical Sciences, Thandalam, Chennai, 602105 Tamil Nadu India; 3 Department of Artificial Intelligence and Data Science, St.Joseph’s College of Engineering, Old Mamallapuram Road, Chennai, 600 119 Tamil Nadu India; 4 Department Electronics and Communication Engineering, Sathyabama Institute of Science and Technology, Chennai, 600119 Tamil Nadu India; 5 Manipal Institute of Technology Bengaluru, Manipal Academy of Higher Education, Manipal, India; 6 Department of Computer Science and Engineering, Kongunadu College of Engineering and Technology, Trichy, 621215 Tamil Nadu India

**Keywords:** Internet of things, Blockchain, Secure communication, Adaptive and sparse attention, Dense long short-term memory, Sorted fitness-based addax optimization algorithm, Optimal key-based elliptic galois cryptography, Engineering, Mathematics and computing

## Abstract

This work presents a secure data transmission process in the Internet of Things (IoT). Initially, the required data are collected and given to the Adaptive and Sparse Attention-based Dense Long Short-Term Memory (ASA-DLSTM) network for intrusion detection. The adaptive nature of the model allows for optimizing the parameters using the Sorted Fitness-based Addax Optimization Algorithm (SF-AOA). Once intrusions are detected, the data is used for the data transmission phase. It is performed using Optimal Key-based Elliptic Galois Cryptography (OK-EGC). By combining Elliptic with Galois fields and an optimal key management strategy, the proposed OK-EGC method enhances both encryption efficiency and security. Moreover, the integration of optimal key-based management using the same SF-AOA ensures that cryptographic keys are dynamically optimized based on the network’s security requirements. Then, the effectiveness of the model is compared with existing systems. The accuracy of the implemented SF-AOA-ASA-DLSTM technique is 95.97%, which is higher than the conventional techniques, such as DNN (83.77%), SVM (83.19%), 1DCNN (90.26%), and ASA-DLSTM (93.6%) for the batch size value 64. Thus, the results display that the designed model addresses the critical challenges of IoT data security by providing both robust intrusion detection and secure communication.

## Introduction

In recent digital networking, secure data transmission is crucial for protecting sensitive data against unauthorized access^1^. Secure data transmission involves various strategies and methodologies aimed at securing data as it moves from one point to another^2^. By ensuring the safety of data in transmission, secure methods guarantee that sensitive details remain confidential and are only available to authorized users or systems. This secured framework is vital for identifying unauthorized users, which is key to maintaining the privacy of sensitive data^3^. The security of the IoT is important, which encompasses a range of practices and technologies aimed at protecting IoT devices and the networks that facilitate their communication^4^. The integration of blockchain technology with IoT highly improves the security of data transmission^5^. Blockchain provides a decentralized and transparent framework that ensures the authenticity and integrity of the data shared among IoT devices^6^. By utilizing blockchain, it becomes feasible to develop privacy-preserving measures, such as encryption and access control strategies, which are essential for protecting sensitive data during both transmission and storage^7^.

One of the main aspects of using blockchain technology is its transparency^8^. Authorized users can access and validate the data stored on the blockchain, allowing them to monitor its authenticity and integrity throughout its entire lifecycle. This feature improves trust and responsibility, as stakeholders individually verify historical data^9^. However, blockchain also has its limitations, particularly in terms of transaction speed and scalability^10^. As a result, the traditional processing speed of blockchain transactions is very low, potentially causing delays and inefficiencies in data transmission^11^. In the blockchain framework, each transaction is accepted as a block, and these blocks are connected to create a continuous chain, which forms an immutable and verifiable transaction record^12^. This capability is essential for preserving data integrity, especially in contexts where the authenticity of the data is crucial. To overcome the issues related to transaction speed and scalability, blockchain is integrated with deep networks.

Deep learning improves data processing and analysis, allowing systems to handle larger volumes of data more effectively^13^. Often, developers prioritize the features and performance of IoT devices over their security. Insufficient security measures during data transmission lead to significant risk during transmission^14^. Many secure transmission methodologies utilize encryption process to protect data, particularly sensitive data^15^. This process transforms data into an unreadable format for unauthorized persons while providing access to those with the appropriate decryption keys^16^. The huge amount of data transmitted by multiple IoT devices can consume network resources, leading to potential delays or the loss of data packets. A huge amount of IoT devices consume battery power, which reduces the processing abilities of the system and may limit their efficiency in handling complex data transmission activities^17^. To tackle the security issues associated with data transmission in IoT, a novel cryptographic approach is proposed. This strategy seeks to improve data security as it traverses an IoT blockchain. By incorporating advanced cryptographic techniques and robust authentication measures, the integrity and confidentiality of the data are maintained, which reduces the risks faced in the IoT system.

### Motivations of the proposed work

The high growth of IoT has led to the deployment of numerous interconnected systems across critical domains, like intelligent transportation systems, smart cities, and so on. Continuously, these systems generate and send huge amounts of sensitive data over heterogeneous networks. Hence, the IoT environments have become highly susceptible to cyber attacks. The conventional security methods are ineffective for IoT devices for numerous reasons. Initially, the majority of the IoT systems are resource-limited, demanding more memory and computational resources. It makes the heavyweight cryptographic models impractical. Further, the existing intrusion detection approaches are mostly developed for static networks. However, the IoT traffic is relatively heterogeneous and dynamic. It results in inaccurate detection accuracy. Also, the conventional cryptographic approaches depend on static key management. It is relatively susceptible to key compromise issues. Though the existing deep learning-aided intrusion detection approaches are powerful, these approaches suffer from poor interpretability and computational complexity problems. These problems motivate the implementation of the designed OK-EGC and ASA-DLSTM combined with blockchain. This framework resolves the intrusion identification, key optimization, secure communication, and trust management in an effective model. The suggested technique is developed to address the gap in the high security demands of IoT devices. Thus, the designed model allows scalable and reliable data transmission.

**Contributions of the proposed model**:

- A secure IoT transmission using a blockchain model is developed for secure data transmission. This framework highlights the importance of utilizing optimal keys, which are essential for reducing risks related to key distribution. A robust key management approach highly improves the security framework of IoT systems. By incorporating blockchain technology, which is decentralized and immutable in nature, an extra layer of security and reliability is provided for data exchange. This integration aids in protecting against data tampering and unauthorized access to confidential data.
- An ASA-DLSTM network is specifically designed for the purpose of detecting intrusions. LSTM networks are exceptionally well-suited for the analysis of time-series data, which often arises in the communication patterns of IoT devices. This capability allows LSTMs to effectively recognize trends and anomalies over time. The inclusion of a sparse attention mechanism in the ASA-DLSTM model allows it to focus on the most pertinent features of the data. This designed approach improves the precision of intrusion detection and reduces the computational resources needed, leading to faster and more efficient analysis. Additionally, the ASA-DLSTM network supports real-time detection and rapid responses to security threats, ensuring the continuous protection of IoT systems against intrusions.
- An OK-EGC methodology is developed to ensure that data identified during intrusion activities is not only encrypted effectively but also transmitted securely, thus boosting the overall security architecture of the system against potential threats. This method employs the principles of elliptic curves to develop a public-key cryptography framework. The advantage of Elliptic Curve Cryptography (ECC) helps to deliver robust security by utilizing smaller key sizes compared to conventional cryptographic systems. This approach is specifically designed to reduce cryptographic attacks. The solid mathematical principles underlying elliptic curves and Galois fields significantly improve the security of the encrypted data, making it significantly more challenging for unauthorized individuals to manipulate the data during its transmission.
- An SF-AOA approach is designed to efficiently optimize parameters and perform key management. The proposed SF-AOA also ensures effective management of cryptographic keys. This implies that the algorithm can make real-time adjustments to encryption keys based on the network’s security demands. If there is a rise in threat levels or the system is subjected to an attack, the SF-AOA can adjust key parameters or select more robust keys to improve security. This integration of adaptive parameter optimization and dynamic key management plays a crucial role in improving data confidentiality.

### Organization

The overall layout of the proposed scheme is provided below. Section  2 presents a review of existing studies and techniques concerning secure data transmission in IoT. Section  3 offers a thorough examination of the suggested secure data transmission model, which incorporates both blockchain technology and cryptographic techniques. In Sect.  4, a detailed explanation of the adaptive deep network framework that aims to detect possible security concerns in IoT systems is outlined. Section  5 discusses the key management approaches utilized within the cryptographic framework in depth. Section  6 includes the experimental analysis of the proposed framework. Finally, Sect.  7 provides a summary of the suggested work.

## Literature survey

### Related works

In 2023, Manjit et al.^18^. have proposed an Elliptic Galois Cryptography (EGCrypto) for providing robust security and improving operational efficiency when compared to conventional cryptographic techniques. This low-complexity strategy allowed EGCrypto to offer substantial security without placing a significant computational strain on resource-limited IoT devices. To improve the performance of EGCrypto, a sophisticated optimization strategy was applied, which included the zoning evolution of control attributes and a self-adaptive differential evolution algorithm based on adaptive mutation. The practical capability of the EGCrypto model was ensured through experimental studies, which demonstrated its superiority over other existing models.

In 2023, Wang et al.^19^ have developed a Blockchain-based Intelligent Healthcare System (BC-IHS) to improve the transmission of medical data. This system was developed to improve the management and communication of healthcare data by utilizing blockchain technology. Its objectives included reducing both the Age of Information (AoI) and energy usage during data transfers while protecting the security of healthcare data. The proposed mechanism’s effectiveness was confirmed through experiments, successfully tackling the significant challenges encountered in the management of healthcare data.

In 2023, Karim et al.^20^ have proposed a novel Blockchain-based Secure Data Exchange Scheme in the Internet of Vehicles (BSDCE-IoV) that was based on the ECV algorithm. This solution was designed to eliminate various potential attacks threatening the IoV environment. A thorough examination was conducted using the real random oracle model, along with an informal security analysis, which validated the scheme in terms of security and privacy. The results indicated that BSDCE-IoV demonstrated superior performance in terms of security and delay compared to some recent works in IoV security.

In 2023, Sutradhar et al.^21^ have suggested a Blockchain-based Zero-Trust Security Model (BC-ZTSM) for the healthcare sector within a fog-cloud computing structure. The BC-ZTSM framework was designed to enforce rigorous security protocols by prioritizing the zero-trust principle. The integrity and confidentiality of the data were preserved through refined cryptographic methods, including a Quad Merkle tree configuration and zero-knowledge proof encryption, which facilitated data verification without disclosing the original content of the data. Thus, the suggested scheme improved the integrity of medical data within edge-fog cloud settings, particularly as healthcare technologies advanced.

In 2023, Alireza et *al*^22^. have introduced an effectual network that ensured secure data transmission in IoT-enabled healthcare systems. This newly developed scalable blockchain architecture utilized the Zero Knowledge Proof (ZKP) mechanism. The architecture was designed to facilitate secure data transfer by protecting sensitive data. Moreover, this system integrated a smart contract that improved data security and ensured safe transaction regulation.

In 2023, Jiang et al.^23^ have developed a bloom filter-based multi-keyword search protocol for ITS to improve efficiency while protecting user privacy. This protocol involved using a bloom filter that identified low-frequency keywords from a set of keywords provided by the ITS data owner. This methodical selection allowed the bloom filter to exclude a substantial portion of ITS data from the search outputs, thereby reducing the computational load. Thus, the suggested scheme was significant for ensuring that data remained accurate in the dynamic environments of ITS.

In 2023, Sonkamble et al.^24^ have introduced a Patient-Centered Healthcare Data Management (PCHDM) system to address access control and privacy concerns based on patient records. The framework successfully integrated the Interplanetary File System (IPFS) and blockchain technology to optimize the storage conditions for EHRs. This decentralized strategy provided robust protection for sensitive health data while granting greater authority to patients regarding who could access their records.

In 2023, Stergiou et al.^25^ have suggested a Cloud-based Monitoring System (CB-MS) to perform virtually without sacrificing the capabilities of conventional monitoring systems. The proposed scheme was achieved by exploring the patterns of the IoT that could improve the efficiency of monitoring systems, especially when deployed over a system hosted in the Cloud. An algorithmic strategy was suggested for the effective transmission and processing of video and image data through the cloud to ensure uninterrupted tasks without any delay.

### Critical analysis and research gaps

Though recent works have provided specific improvements in enhancing the security of the IoT via cryptographic approaches, blockchain models, and effective data management methods, some crucial research gaps remain.

Manjit et al.^18^ have presented EGCrypto and illustrated its efficiency in offering better security with minimized computational burden. Nevertheless, this work mainly concentrated on the efficiency of the encryption and hasn’t considered an effective intrusion detection approach. As an outcome, this approach secures the data only after transmission. But this model becomes susceptible to pre-transmission attacks. Wang et al.^19^, Karim et al.^20^, Sutradhar et al.^21^, and Alireza et al.^22^ have utilized blockchain mechanisms for exchanging data securely in different domains. These mechanisms have poor intelligence, making them ineffective against zero-day attacks and dynamic intrusion features monitored in the IoT networks. In addition, the majority of the blockchain-aided models face communication and computational overhead. It limits the practicality of the existing approaches. Jiang et al.^23^ and Sonkamble et al.^24^ have concentrated on improving the efficiency of the data search and access control employing bloom filters and the integration of IPFS-blockchain. Though these works improve the storage efficiency and data management, these models haven’t resolved the issues of secure key management and real-time intrusion detection. Stergiou et al.^25^ have designed a cloud-assisted monitoring approach that improved the processing efficiency and data transmission. Nevertheless, this approach highlights performance optimization rather than security intelligence.

From the above critical analysis, the following research challenges are identified and addressed by the designed mechanism.

- The majority of the conventional works concentrate on blockchain and encryption. However, these models avoided the AI-based attack detection. To address this issue, the suggested work presents ASA-DLSTM-based intrusion detection. This model effectively captures the evolving attack features.
- The existing cryptographic models utilize fixed keys. It makes them susceptible to replay threats and key compromise issues. To rectify this problem, this work introduces SF-AOA for the dynamic key optimization process. It guarantees reliable cryptographic security.
- The conventional deep learning mechanisms do not utilize the sparse attention method. It results in minimized efficiency and redundant processing. To address this problem, this work presents a sparse attention method for minimizing the computational overhead and feature redundancy.
- The previous works consider intrusion detection, trust management, and encryption as individual operations. It minimizes the performance rate of the models. Unlike existing methods, the designed framework provides a fully combined and lightweight security solution, thus achieving a high performance rate.

### Problem statement

Secure data transmission is crucial for protecting sensitive data from cyber-attacks. Blockchain technology is a transparent framework that transmits data among IoT devices for the access of healthcare organizations. It provides a decentralized and exchangeable platform to transmit data in real-time. The cryptographic techniques in the blockchain may protect the data from unauthorized access by a third party. The major advantage of the blockchain mechanism is that it cannot be deleted or tampered with. Despite this, the existing models faced major challenges when transmitting data among different organizations. The problem associated with blockchain data transmission includes a wide range of scalability issues and vulnerability to attacks. Moreover, it has difficulty in handling the massive volume of data transmission. Hence, an advanced technique is developed in this work to eliminate the potential problems in the existing models. Table 1 gives the features and challenges involved in existing blockchain-based secure data transmission models.

- A major problem that evolved in the existing models is security vulnerabilities. Because blockchain transactions are transparent, it makes it easy for attackers to steal sensitive data. These security flaws can lead to unauthorized access by third parties. It can be addressed by the deployment of effective deep learning techniques. The development of the deep learning model can provide high security by identifying abnormal patterns that may indicate security flaws and provide strong data privacy.
- One of the major concerns in data transmission over blockchain is its scalability issues. As the number of transmissions increases on the network, it leads to a slower response to transactions, which leads to higher latency. To overcome this challenge, an implementation of an effective adaptive optimization algorithm is suggested. By dynamically adjusting the parameter, the system can handle data loads, which helps to reduce high latency.
- Another challenge related to the blockchain-based data transmission is key duplication. It is caused by the usage of the same key values among users, which leads to data corruption. To overcome this challenge, a cryptography method can be suggested. It provides private keys for each user by connecting to the blockchain using a cryptography function that can secure data transmission.
- The traditional methodologies for secure data transmission using blockchain face vulnerable cyber attacks, which affect the integrity of the network. This challenge can be overcome by performing an optimal key selection process. It works by ensuring only the authorized parties have access to the data. These keys protect the security of the data, which contributes to data integrity during data transmission.

**Table 1:** Features and challenges of the existing blockchain-based secure data transmission models.

Author [citation]	Methodology	Features	Challenges
Manjit *et el*. [18]	EGCrypto	• This model can secure communication in IoT devices, ensure the safe transfer, and easily recover encrypted data. • The enhanced computational speed of the EGCrypto model indicates an effective execution in securing IoT networks.	• It is easily exposed to attacks due to the compatibility issue, which can affect overall system security. • To strengthen resilience against vulnerable attacks, further deployment of advanced optimization techniques is required.
Xiaojie Wang *et el*. [19]	BC-IHS	• It achieves high accuracy in data transmission. • It can minimize the consumption of energy during the transmission of data.	• Ensuring the integrity of data in the blockchain is challenging.
Karim *et el*. [20]	SDCE-IoV	• It can prevent Man-in-the-Middle and physical vehicle capture attacks. • It can handle high data dimensions even with minimal computational and communication complexities to protect security.	• It can be optimally designed and implemented to develop a flexible and practical vehicular network. • Network latency often leads to security vulnerabilities.
Sutradhar *et el*. [21]	BC-ZTSM	• It can address scalability issues in a large-scale environment. • Due to the protected function of the model, it provides high data integrity.	• It leads to vulnerability to attacks when the private keys are compromised.
Alireza Jolfaei *et el*. [22]	BDSDT	• It can protect data from unauthorized access due to the presence of a transparent platform. • It improves the security of data transmission and reduces data breaches.	• The speed of data transmission can be slow due to the scalability issue. • Handling huge amounts of data potentially causes storage challenges.
Jiang *et el*. [23]	BC-ITS	• It helps to improve search efficiency on the blockchain. • It can secure user data, reducing the risk of manipulation.	• Providing integrity and privacy in the ITS dataset requires a cryptographic-based primitive guaranteeing integrity. • It can lead to congestion during high-throughput data transmission.
Sonkamble *et el*. [24]	BC-EHR	• It strengthens security by providing a decentralized framework. • It can identify poor access control of sensitive data.	• Computational cost is high. • To protect patient data, data privacy protocols are needed.
Stergiou *et el*. [25]	CB-MS	• This model offers a safe system for data transmission.	• Due to the use of various data formats from healthcare organizations, it is difficult to exchange data across blockchain networks.

## Introduction of a developed secure data transmission model in IoT using blockchain technology with cryptography

### Architectural view of the implemented secure IoT framework

**Fig. 1:**
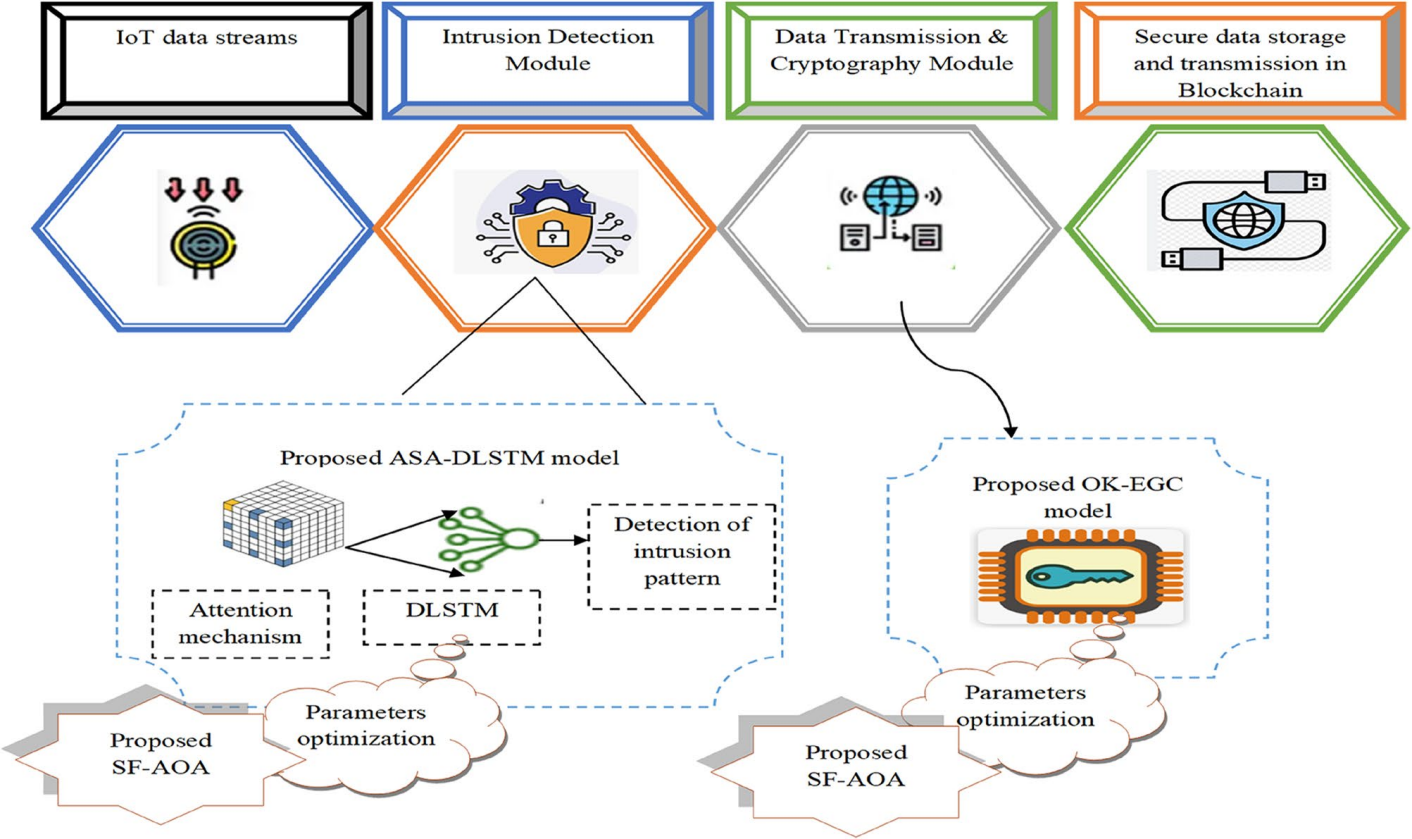
Architectural view of the implemented secured IoT framework.

Blockchain networks encounter considerable scalability challenges as the volume of IoT devices grows. The capacity for processing transactions is limited by block size and the time needed to reach consensus, potentially constraining the implementation of large-scale IoT solutions. Furthermore, the time taken for confirming transactions and achieving consensus in blockchain systems leads to delays; this latency can adversely affect both performance and reliability. By tackling these issues, a novel framework is developed. The architectural view of the implemented secure IoT framework is provided in Figure 1.

### Methods description

The developed blockchain-based secure data transmission approach is implemented to provide a systematic determination for intrusion detection and secure data transmission in IoT platforms. The procedure for this architecture is provided in the following detailed step-by-step process.

**Step 1: Data Collection from IoT Devices**.

- This developed approach is undertaken with the collected data from IoT devices, and the proposed framework starts the data collection from diverse IoT devices. This data forms the strength of the intrusion detection system and consists of time-series data produced by the devices.

**Step 2: Intrusion Detection Using the ASA-DLSTM model**.

- The garnered data is processed by the ASA-DLSTM network. This model integrates the advantages of LSTM networks, which excel at analyzing sequential data. The ASA-DLSTM improves detection efficiency through a sparse attention mechanism that highlights the most pertinent features of the input data. This targeted approach not only increases detection accuracy but also minimizes the computational load, facilitating real-time analysis. Moreover, the model is capable of adapting to the ever-evolving nature of potential cyber threats, allowing for prompt detection and response to intrusions. The ASA-DLSTM improves detection efficiency through a sparse attention mechanism that highlights the most pertinent features of the input data. This targeted approach not only increases detection accuracy but also minimizes the computational load, facilitating real-time analysis. Moreover, the model is capable of adapting to the ever-evolving nature of potential cyber threats, allowing for prompt detection and response to intrusions.

**Step 3: Parameter Optimization Using SF-AOA Algorithm**.

- The fine-tuning process is carried out by the SF-AOA algorithm to enhance the detection accuracy. To ensure optimal detection performance, the ASA-DLSTM network employs SF-AOA for tuning its parameters. This adaptive strategy ensures that the model’s effectiveness progresses with new emerging threats, thereby improving the overall accuracy and Critical Success Index (CSI) of the security framework.

**Step 4: Intrusion Responses to Secure Data Transmission**.

- Once the intrusion is detected, the system adjusts a secure transmission stage to prevent sensitive data from being transmitted.

**Step 5: Secure Data Transmission Using the OK-EGC technique**.

- The developed method presents an OK-EGC technique for secure data transmission. By combining elliptic curve cryptography with Galois fields, the OK-EGC technique improves both the efficiency of encryption and the security level.

**Step 6: Key Management Strategy Using SF-AOA**.

- A crucial aspect of the OK-EGC method is its optimized key management strategy, which is also fine-tuned through SF-AOA. This dynamic modification of cryptographic keys aligns with the security requirements of the network, significantly improving data confidentiality. Ultimately, the framework achieves secure data transmission.

**Step 7: Secure Data Storage in Blockchain**.

- After secure data transmission, the data is stored in a blockchain, which prevents it from unauthorized access.

**Step 8: Efficacy Validation**.

- The experimental validation is conducted using diverse traditional approaches and standard datasets. The model’s effectiveness is assessed against existing systems, illustrating that it tackles crucial challenges related to IoT data security. It provides strong intrusion detection capabilities along with secure communication channels, ensuring comprehensive protection for IoT networks.

### Dataset collection from IoT

The data needed for this proposed framework are gathered from two relevant datasets, and the descriptions of these datasets are provided below.

Dataset 1 (APA-DDoS) is an essential resource for researchers and developers focused on improving cybersecurity strategies. The data is collected from the link https://www.kaggle.com/datasets/yashwanthkumbam/apaddos-dataset accessed on 2025-04-24. This dataset is organized to support the proposed models, providing a wide range of detailed objects of malicious traffic. Such variations are crucial for training machine learning algorithms to identify not only abnormal traffic that signals potential attacks but also typical traffic behaviors.

Dataset 2 (kddcup99.csv) serves as a prominent dataset in the realm of network intrusion detection. The data is collected from the link https://www.kaggle.com/datasets/venkatakanumuru/kddcup99csv accessed on 2025-04-24. This dataset is based on simulated network traffic, reflecting different kinds of activity within a military network context. It encompasses not only typical connections but also a range of attack scenarios.

The data collected from these datasets is indicated as $W{S_k}$, where $k$ is the total amount of collected data.

### Blockchain-based IoT security

Blockchain-based security for IoT involves utilizing blockchain technology to improve the safety of IoT devices and networks. As the number of IoT devices starts to increase and their roles expand across diverse applications, the demand for strong, secure communication and data integrity in these environments becomes essential. Blockchain guarantees that data recorded on its ledger remains unchangeable and resistant to tampering. Each communication performed by IoT devices is documented as a block within the blockchain, rendering it nearly impossible to modify previous transactions without the agreement of the network. This capability is significant for preserving the accuracy of sensor data and records.

Moreover, blockchain supports secure authentication of devices via cryptographic techniques. Each IoT device can possess a different digital identity stored on the blockchain, which facilitates secure verification and aids in preventing unauthorized access. Additionally, blockchain develops a reliable framework for data sharing among IoT devices, allowing them to exchange data directly and securely without depending on a central server. This is especially significant in situations such as medical devices exchanging patient data or connected vehicles communicating traffic data. Therefore, blockchain-based IoT security provides a promising method for addressing the various vulnerabilities present in IoT systems for secure authentication.

## Adaptive deep network-based intrusion detection framework for detecting security vulnerabilities in an IoT environment

### Sparse attention-based dense LSTM description

To improve the reliability of the intrusion detection process, an effective SA-DLATM strategy is implemented. This network integrates the features of both DLSTM and sparse attention, which improve the sequential data processing. Dense LSTM architecture is more efficient than the conventional framework, which plays a vital role in handling sequential data due to its capability to retain long-term dependencies through its specialized gating mechanisms.

Forget gate is mathematically expressed in Eq. (1).

1 $${G_f}=\varphi \left( {R_{p}^{k} \times {D_{k - 1}}+H_{p}^{k} \times {a_k}+{m_p}} \right)$$

Here, the weight matrices are indicated as$R_{p}^{k}$and$H_{p}^{k}$. The output from the previous state is indicated as${D_{k - 1}}$, and the term ${a_k}$is the current input data at the time step $k$. The bias term for the forget gate is indicated as${m_p}$.  

The input gate is provided in Eq. (2).

2 $${G_I}=\varphi \left( {R_{i}^{k} \times {D_{k - 1}}+H_{i}^{k} \times {a_k}+{m_i}} \right)$$

Candidate gate is derived in Eq. (3).

3 $${\tilde {W}_c}=\tan k\left( {R_{c}^{k} \times {D_{k - 1}}+H_{c}^{k} \times {a_k}+{m_c}} \right)$$

The cell state is improved by utilizing the outputs from the gates using Eq. (4)

4 $${W_c}={G_f} \times {W_{c - 1}}+{G_I} \times {\tilde {W}_c}$$

The output gate determines what data will be passed on to the next stage of the sequence using Eq. (5).

5 $${G_o}=\varphi \left( {R_{o}^{k} \times {D_{k - 1}}+H_{o}^{k} \times {a_k}+{m_o}} \right)$$

6 $${O_T}={G_o} \times \tan k\left( {{W_c}} \right)$$

The dense network is connected to the LSTM to further improve the features generated by the LSTM. It consists of two dense layers, which refine the output obtained from the LSTM to improve performance in intrusion detection tasks. Dense layers contribute to significant model complexity, which is susceptible to overfitting, particularly when training data is limited. Additionally, Dense LSTMs experience low convergence during training due to the large number of parameters and their complex nature. It also leads to an increase in the parameter count, demanding high computational power and extended training durations. To address these issues, a sparse attention network is usually integrated following the LSTM layer and preceding the dense layers within the network structure. This setup allows the network to emphasize essential data while minimizing the dimensionality of the LSTM outputs before they are fed into the dense layers for intrusion detection.

The sparse attention mechanism evaluates attention scores based on the significance of the input data, concentrating on the important features captured from the LSTM. It effectively eliminates noise and redundancies in the sequential data. By utilizing attention mechanisms, the network can flexibly modify the weights assigned to different time steps in the input sequence, thus allowing the model to retain critical context while eliminating non-essential details. For a sequence of length $L$, the attention matrix ${A_{tn}}$ is calculated using Eq. (7).

7 $${A_{tn}}=\sum\limits_{{e \in {F_i}}} {{V_{gh}}{B_p}}$$

**Fig. 2:**
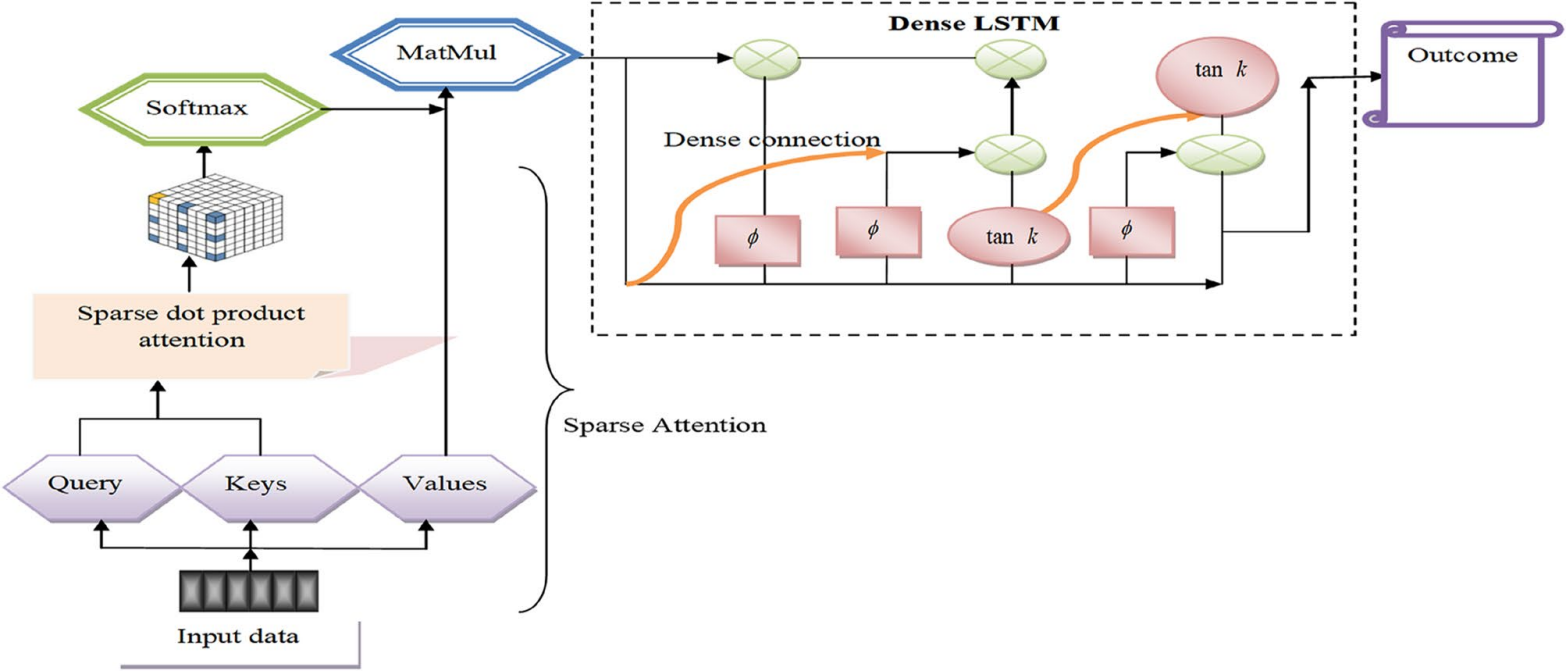
The gating mechanism of the SA-DLATM model.

Here, the weighted sum of the values ${B_p}$is multiplied by their corresponding attention scores${V_{gh}}$. This sparsity allows the SA-DLATM to focus on the most relevant parts of the input data, improving efficiency. It improves the network’s efficiency and improves the overall recognition accuracy and convergence speeds of the network by applying dense layers with more pertinent features. The network mechanism of the SA-DLATM model is visually represented in Fig. 2.

### ASA-DLSTM for intrusion detection

**Fig. 3:**
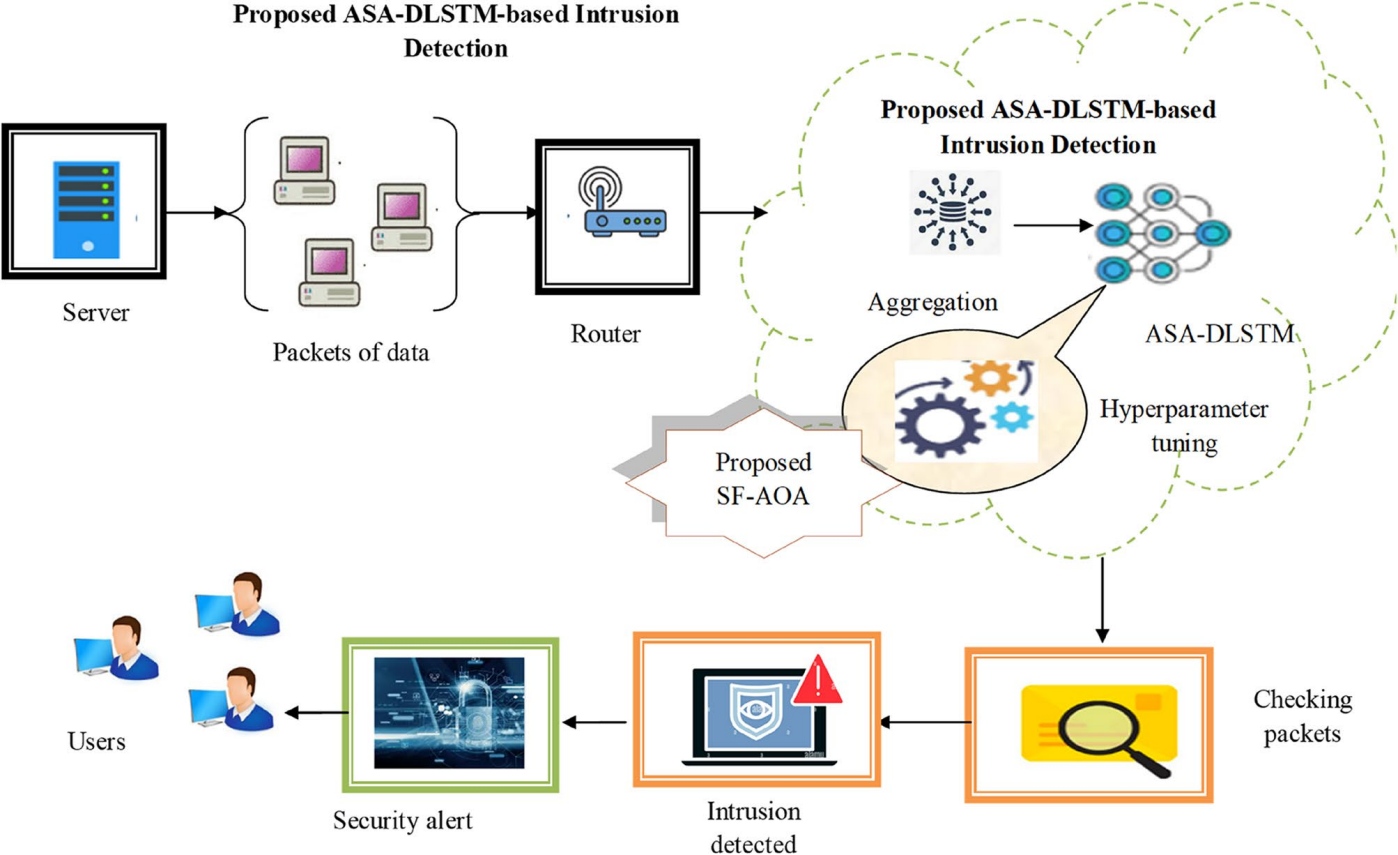
Diagrammatic view of proposed ASA-DLSTM-based intrusion detection.

The proposed ASA-DLSTM network is a sophisticated framework aimed at improving the detection of intrusions within network environments, as shown in Fig. 3. This model starts by collecting network data, comprising various attributes visible in network packets. The data is fed to ASA-DLSTM for the detection of intrusion. Within the ASA-DLSTM design, the LSTM element plays a vital role in inserting time-series data. LSTMs, a variant of RNN, are designed to effectively recognize patterns over sequential data. They are capable of retaining data from earlier time points while eliminating unwanted details, making them particularly adept at detecting intrusions that may have time-based dependencies, such as recurring patterns over time. The self-attention model allows the model to concentrate on specific input data that holds greater significance for intrusion detection. It gives different levels of importance to various features according to their relevance to the task. By evaluating attention scores, the ASA-DLSTM emphasizes key data points while framing other data in context, resulting in more precise and reliable identification of anomalies or intrusions. The adaptive feature of the ASA-DLSTM model allows it to evolve its learning strategies based on the features and patterns of input data. This allows the ASA-DLSTM to continuously learn and adjust from real-time network inputs, improving its accuracy and CSI of the system. The objective function is mathematically expressed in Eq. (8).

8 $${R_{obj}}=\mathop {\arg \hbox{min} }\limits_{{\left\{ {Er_{h}^{{DLSTM}},Rr_{k}^{{DLSTM}},Ec_{{jj}}^{{DLSTM}}} \right\}}} \left( {\frac{1}{{Ar+CSI}}} \right)$$

Here, the term $Er_{h}^{{DLSTM}}$is the optimized neuron counts that vary in the range of$\left[ {5 - 225} \right]$, $Rr_{k}^{{DLSTM}}$is the optimized learning rate that varies in the range of$\left[ {0.01 - 099} \right]$, and $Ec_{{jj}}^{{DLSTM}}$is the optimized epoch count that varies in the range of$\left[ {5 - 50} \right]$. The formulas for calculating accuracy$Ar$ and CSI values are provided in Eq. (9) and Eq. (10).

9 $$Ar=\frac{{\left( {Wve+Wne} \right)}}{{\left( {Wve+Ype+Wne+Yne} \right)}}$$

10 $$CSI=\sum\limits_{{p=1}}^{n} {\beta W{S_k}}$$

Here, the true positive and true negative values are indicated as $Wve$and$Wne$. The false positive and false negative values are indicated as $Ype$and$Yne$, respectively. The term $p$ is the individual factors, and $\beta$ is the weights assigned to the factors. After its training phase, the ASA-DLSTM model is capable of effectively observing network activity. It analyzes input data in real-time, leveraging the insights gained from the LSTM and self-attention layers to identify suspicious patterns in the network.

#### Novelty of ASA-DLSTM-based intrusion detection

The novelty of the designed ASA-DLSTM is its capacity to concentrate on the highly relevant IoT features and optimize the network parameters. Unlike the existing attention-based or LSTM techniques, the designed ASA-DLSTM utilizes the sparse attention strategy for eliminating the unrelated data. Thus, the designed model minimizes the computational complexity and improves the detection accuracy. In addition, the inclusion of SF-AOA allows the technique to fine-tune its parameters based on the evolving attack features. It makes the designed ASA-DLSTM highly effective for IoT intrusion identification.

### Implemented SF-AOA for parameter tuning

The proposed model utilizes SF-AOA due to its adaptive and effective optimization capabilities, which are inspired by the original AOA. SF-AOA dynamically optimizes parameters within the adaptive model, ensuring these parameters remain robust against evolving attack patterns. Moreover, SF-AOA improves cryptographic key management by continuously adjusting keys based on the network’s current security demands. This dual functionality significantly strengthens both data confidentiality and accuracy, CSI, and overall security of the system. The optimization power of SF-AOA from the foundational principles of AOA, which effectively balances exploration and exploitation phases, is modeled after the addax’s natural foraging and digging behaviors. This balance allows for improved convergence rates and higher-quality solutions, especially in complex search spaces. However, the performance and computational efficiency of AOA in very large-scale problems remain less explored, presenting challenges in such scenarios. While AOA simulates the addax’s behavior to maintain a balance between global search (foraging) and local refinement (digging), achieving the optimal equilibrium in complex landscapes is difficult and demands careful tuning. To address these challenges, the AOA is improved by incorporating a fitness-based sorting strategy, implemented through the following steps:

Sorting Fitness and Solutions: At each iteration, candidate solutions are evaluated, and their fitness values are sorted in ascending order. This ranking allows the algorithm to differentiate between the most promising and weaker solutions.

Selecting Extreme Fitness Subsets: The top five best-performing fitness values indicated as $F{F_k}$ and the bottom five worst ones indicated as$L{F_k}$ are extracted, providing a clear analysis of the population’s quality extremes.

Adjusting the Random Variable: Using these fitness subsets, SF-AOA modifies the random parameter${J_r}$ using Eq. (11).

11 $${J_r}=\frac{{\sum\limits_{{k=1}}^{5} {L{F_k}} }}{{\sum\limits_{{k=1}}^{5} {F{F_k}} }}$$

Here, the term $k$ is the total number of fitness values that vary $\left[ {1 - 5} \right]$. This modification improves convergence speed and solution quality by focusing more on superior solutions while retaining awareness of weaker search regions. As a result, it reduces the risk of premature convergence and improves the algorithm’s capability to navigate complex optimization settings.

#### Novelty of the SF-AOA

**Fig. 4:**
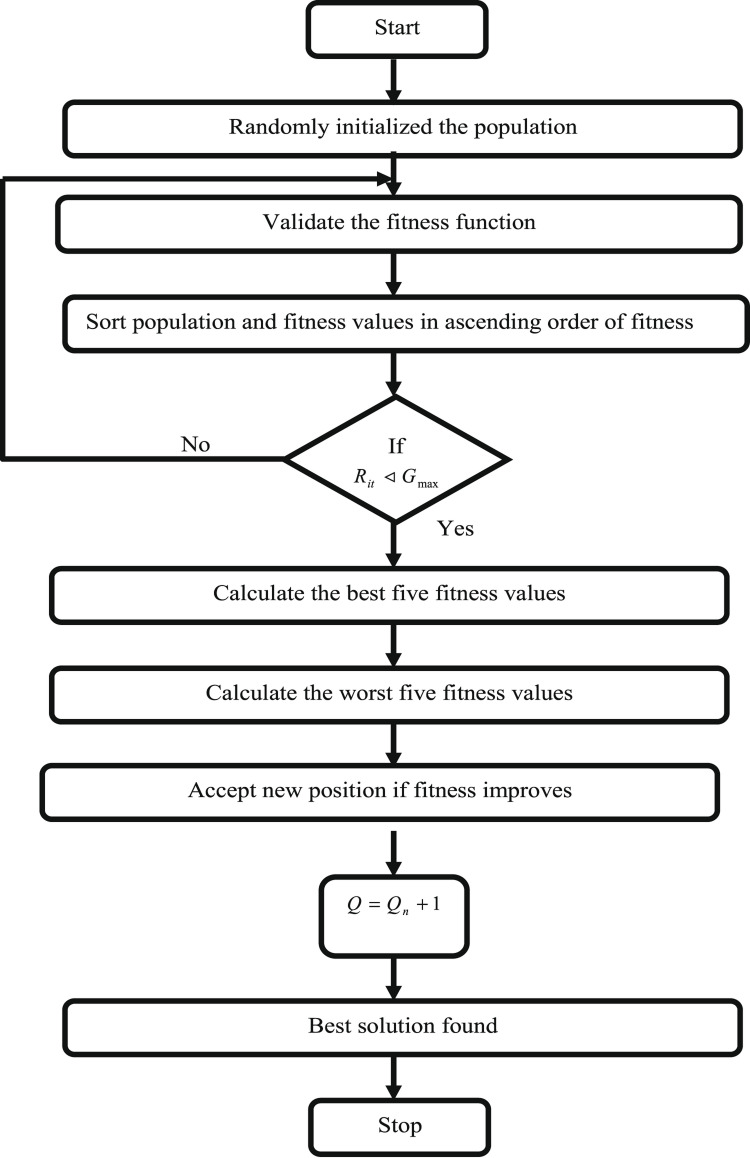
Flow chart of proposed SF-AOA strategy.

The novelty of the designed SF-AOA is its fitness-ranking-based mechanism. Here, the candidate solutions are arranged based on the fitness values to assist the exploration and exploitation very efficiently. Unlike the existing AOA, the designed SF-AOA presents a sorted fitness strategy. This mechanism prioritizes the high-quality outcomes, thus improving the convergence speed. It makes the designed SF-AOA efficient for parameter optimization and key generation in IoT environments. The pseudo-code of the proposed SF-AOA is provided in Algorithm 1, and its flow chart is provided in Fig. 4.



## Optimal key-based cryptography for performing encryption to secure the data transmission in IoT

### Elliptic Galois cryptography technique

In this proposed model, the EGC strategy is employed for secure data transmission, where the cryptographic tasks are explained over the finite Galois fields to support effective data encryption and key generation. The elliptic curve over a finite field is expressed in Eq. (12).

12 $${q^2}={c^3}+h\bmod R+pc$$

Here, the terms $c$ and $q$ are the coefficients that characterized the elliptic curve, $h$ is the parameter that modifies the curve shape. The prime characteristic of the finite field in which the elliptic curve operates is indicated as $R$. The variables $h$ and $p$ take different values, resulting in various elliptic curves. The Galois field is indicated as${F_G}\left( T \right)$, and comprises a finite set of elements using the equations below.

13 $${F_G}\left( T \right)={E_o} \cup {E_1} \cup {E_2}....{E_{m - 1}}$$

    

Where:

14 $${E_o}=\left( {0,1,2...T - 1} \right)$$

15 $${E_1}=\left( {T \cdot T+1,T+2...T+T - 1} \right)$$

16 $${E_{m - 1}}=\left( {{T^{m - 1}} \cdot {T^{m - 1}}+1......{T^{m - 1}}+T - 1} \right)$$

Each factor in ${E_o}$ follows a cyclic order, and each non-zero element includes a multiplicative inverse. It guarantees that division is practical within the domain.

Key Generation Process:

Key generation in ECC involves the creation of a private key and its corresponding public key. The public key${K_p}$ is formed by multiplying the private key${K_{pub}}$ by a generator point $X$ on the elliptic curve, which is mentioned in Eq. (17).

17 $${K_p}=X \cdot {K_{pub}}$$

  

The elliptic curve allows a task for adding points on it. Given two points $v$ and $b$  on the curve, their addition is expressed in Eq. (18).

18 $$E+R=v+b$$

  

The resulting point $\left( {v,b} \right)$ lies on the curve. Thus, ECC provides high security with shorter key sizes when compared with conventional encryption techniques, as the difficulty of solving the discrete logarithm problem on elliptic curves is considerably greater. By utilizing these foundational concepts, EGC effectively safeguards data and encrypts data in the IoT domain, where secure data transmission is of utmost importance.

#### Security significance in EGC

The EGC attains powerful security with smaller key sizes compared to existing cryptographic approaches. By combining the elliptic curve tasks with finite Galois field arithmetic, the designed EGC model guarantees low storage and communication overhead, secure encryption and key generation, and effective computation for resource-limited IoT systems. Hence, the EGC offers an efficient cryptographic basis for secure data transmission in IoT scenarios.

### Optimal key generation using SF-AOA

After detecting intrusions within the IoT network, the system forwards to the data transmission stage, which utilizes OK-EGC to guarantee secure communication. During this phase, cryptographic keys are dynamically created and optimized for both security and performance through the SF-AOA optimization algorithm. The EGC methodology integrates ECC concepts with the mathematical framework of Galois fields, providing robust cryptographic mechanisms that facilitate efficient key generation, encryption, and decryption processes. Compared to traditional asymmetric cryptosystems, EGC significantly lowers computational demands.

Rather than depending on fixed keys, the system generates and manages cryptographic keys dynamically. SF-AOA specifically refines the key generation process by identifying keys that improve encryption robustness while minimizing computational resource usage. It adaptively fine-tunes keys based on the current security setting of the network. This optimization strategy guarantees that the keys maintain strong resistance against attacks while reducing both the execution time and memory footprint associated with cryptographic operations. The optimization objectives are mathematically represented in Eq. (19).

19 $${F_{obj}}=\mathop {\arg \hbox{min} }\limits_{{\left\{ {K{y_h}} \right\}}} \left( {{T_c}+{S_m}} \right)$$

Here, the terms ${T_c}$and ${S_m}$are the time consumption and memory size of the system, which are described below. And also, the term $K{y_h}$is the optimized key in the range of$\left[ {0 - 1} \right]$.

#### Generation of Normalized Value

In this proposed framework, the normalized key was created with the support of the SF-AOA optimization algorithm, where the optimized key lies within the range$\left[ {0 - 1} \right]$. Here, it is not directly applied as a cryptographic key, which is managed as a seed, or it is converted into a secure key with the support of a multi-stage process. At first, this normalized value is extended to a 256-bit integer space by cryptographic hashing using SHA-256 to establish uniform randomness and struggle of statistical attacks. This hash value is mapped by a rational elliptic curve private key with the support of modular reduction. This modular reduction technique establishes that the created keys include cryptographic strength necessities such as 256-bit security, which can enhance the optimization process.

#### Time consumption

The total time required for encryption and decryption with a candidate key.

#### Memory size

The amount of memory utilized during these cryptographic tasks.

For each candidate key, the OK-EGC algorithm performs encryption of the IoT data followed by decryption using the same key. The keys optimized by SF-AOA minimize resource utilization during the cryptographic cycle without sacrificing security. The dynamic key management complicates attempts by adversaries to predict, thus improving confidentiality and data integrity. Incorporating SF-AOA allows the system to respond promptly to intrusion detection, adapting security parameters in real-time. Hence, encryption and decryption operations have become accurate and secure. Thus, the combination of OK-EGC and SF-AOA produces secure, efficient, and adaptive cryptographic keys for data transmission across IoT networks.

#### Novelty of OK-EGC

**Fig. 5:**
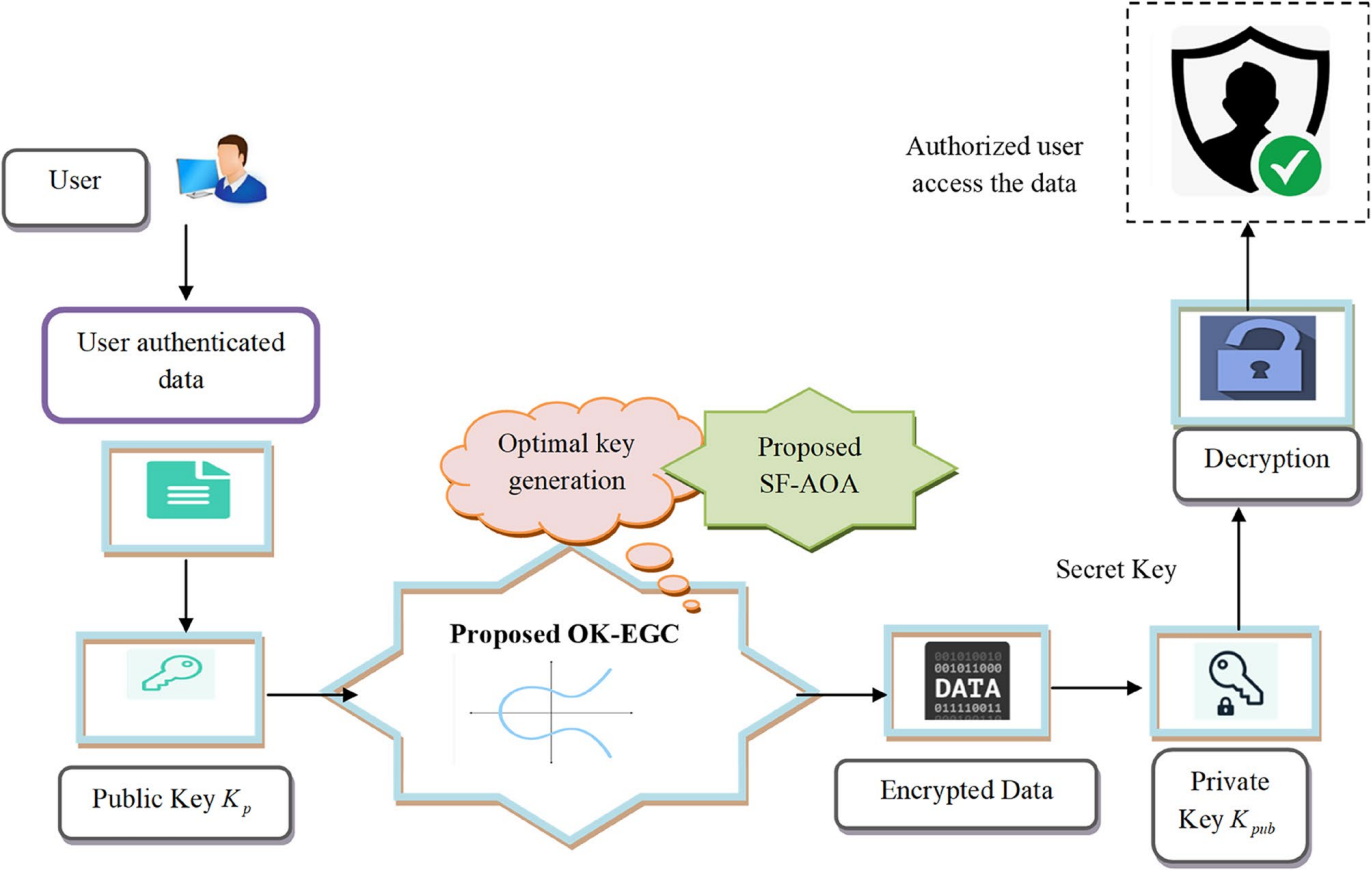
Diagrammatic representation of optimal key generation using SF-AOA.

The novelty of the designed OK-EGC is its hybrid combination of ECC with Galois field tasks and an innovative key optimization process. Unlike the traditional cryptographic approaches, the designed OK-EGC utilizes an SF-AOA-based dynamic key generation process. It allows the cryptographic keys to be optimally selected on the basis of network security demands. It leads to better resistance to cryptographic threats and enhanced encryption efficiency. It makes the designed OK-EGC highly applicable for lightweight IoT security devices. This framework improves secure communication by reducing vulnerabilities and improving overall system performance. Optimal key generation using SF-AOA is diagrammatically given in Fig. 5.

### Secure data storage in blockchain

Secure data storage using blockchain technology involves storing data and protecting it from unauthorized modifications and access. In this system, data is distributed across numerous nodes within a peer-to-peer network, which removes the concern of a single point of failure. This distributed nature improves security by ensuring a single party has full control over the data, thereby decreasing the possibilities of data loss and unauthorized changes.

**Fig. 6:**
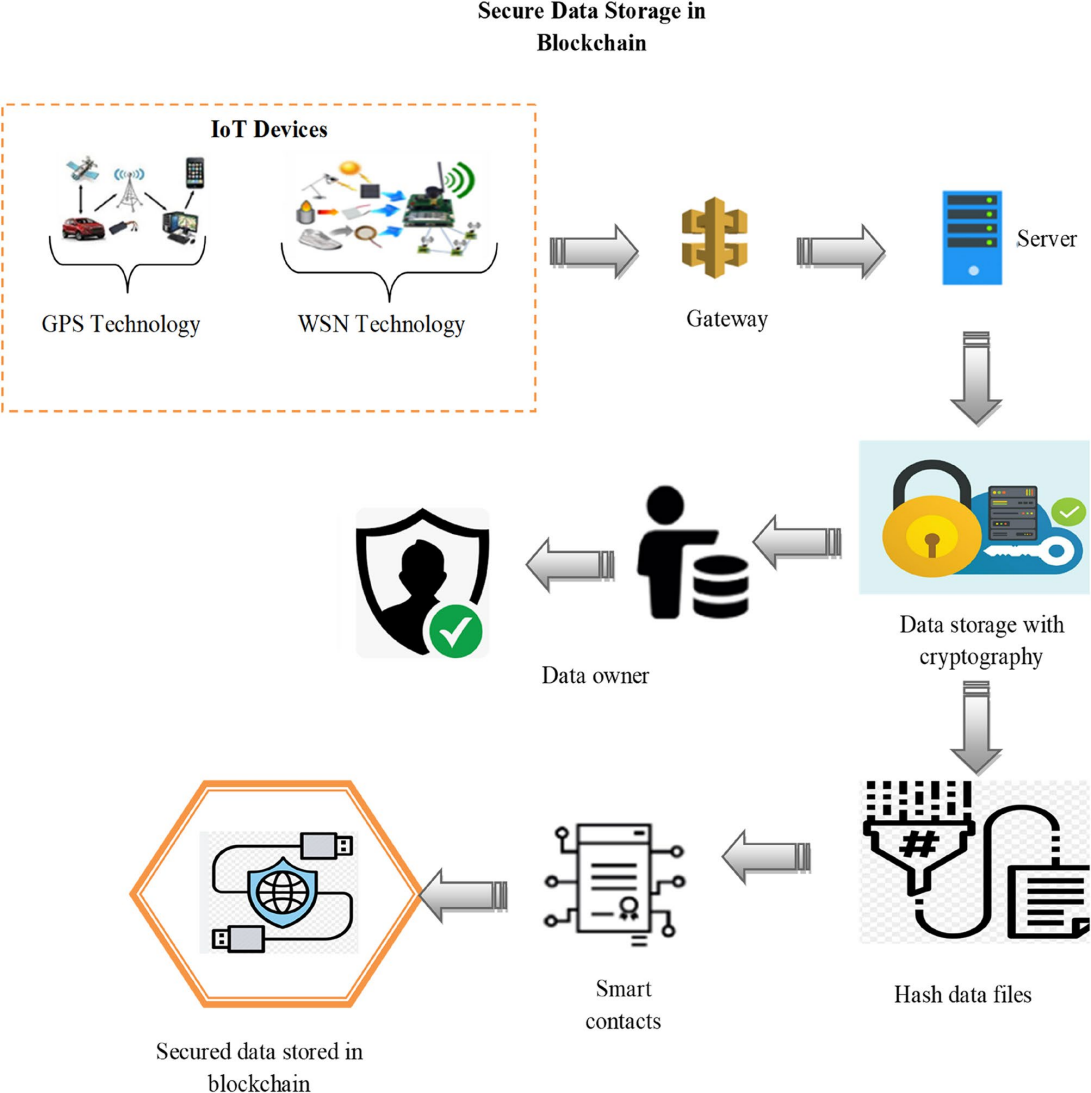
Secure data storage in blockchain.

Once data is stored on the blockchain and validated through a consensus process, it becomes unchangeable and cannot be erased retroactively. This stability is secured by cryptographic hash functions that link blocks together, making any attempts to alter the data obvious and easily traceable. The stored data is protected using cryptographic methods that ensure its confidentiality and integrity. A new transaction is added to the blockchain following agreements between the network users, which help to prevent fraudulent entries. Because the blockchain ledger is copied across all involved nodes, the data remains accessible even if some nodes fail or are attacked. Therefore, blockchain-based secure data storage utilizes its decentralized, immutable, and cryptographically secured framework to create a reliable and tamper-resistant record. The suggested work utilizes the permissioned consortium blockchain for data storage purposes. This approach guarantees strong data integrity, privacy, and availability, positioning blockchain as an effective solution for managing secure data, particularly in settings that require transparency, traceability, and robustness against cyberattacks. Secure data storage in blockchain is visually represented in Fig. 6.

## Results and discussion

### Experimental setup

The proposed blockchain-based secure IoT communication was implemented in Python software. This framework employed the SF-AOA strategy for the optimization process with parameters such as population size of 10, chromosome length of 3, and maximum iteration of 50. The reliability of the proposed blockchain-based secure communication was compared with conventional algorithms and techniques like Red-billed Blue Magpie Optimizer (RBMO)^26^, Pelican Optimization Algorithm (POA)^27^, Frilled Lizard Optimization (FLO)^28,29^, and Addax Optimization Algorithm (AOA)^30^. Conventional techniques like Deep Neural Network (DNN)^31^, Support Vector Machine (SVM)^32^, and One-Dimensional Convolutional Neural Network (1DCNN)^33^, Sparse Attention (SA)^34^, and Dense Long Short-Term Memory (Dense-LSTM)^35^ were utilized to compare the efficiency of the intrusion detection model. The reliability of cryptography was validated over traditional cryptography algorithms like Data Encryption Standard (DES)^36^, Advanced Encryption Standard (AES)^37^, and Elliptic Curve Cryptography (ECC)^38,39^.

### Performance metrics analysis

Some performance metrics used to validate the efficiency of the proposed blockchain-based secured IoT communication are provided in the points below.

  

$$Ar=\frac{{\left( {Wve+Wne} \right)}}{{\left( {Wve+Ype+Wne+Yne} \right)}}$$

  

$$FNR=\frac{{Yne}}{{\left( {Wve+Yne} \right)}}$$

  

$$FNR=\frac{{Ype}}{{\left( {Wne+Ype} \right)}}$$

  

$$precision=\frac{{Wve}}{{\left( {Wve+Ype} \right)}}$$

  

$$specificity=\frac{{Wne}}{{\left( {Wne+Yne} \right)}}$$

### Convergence analysis

**Fig. 7:**
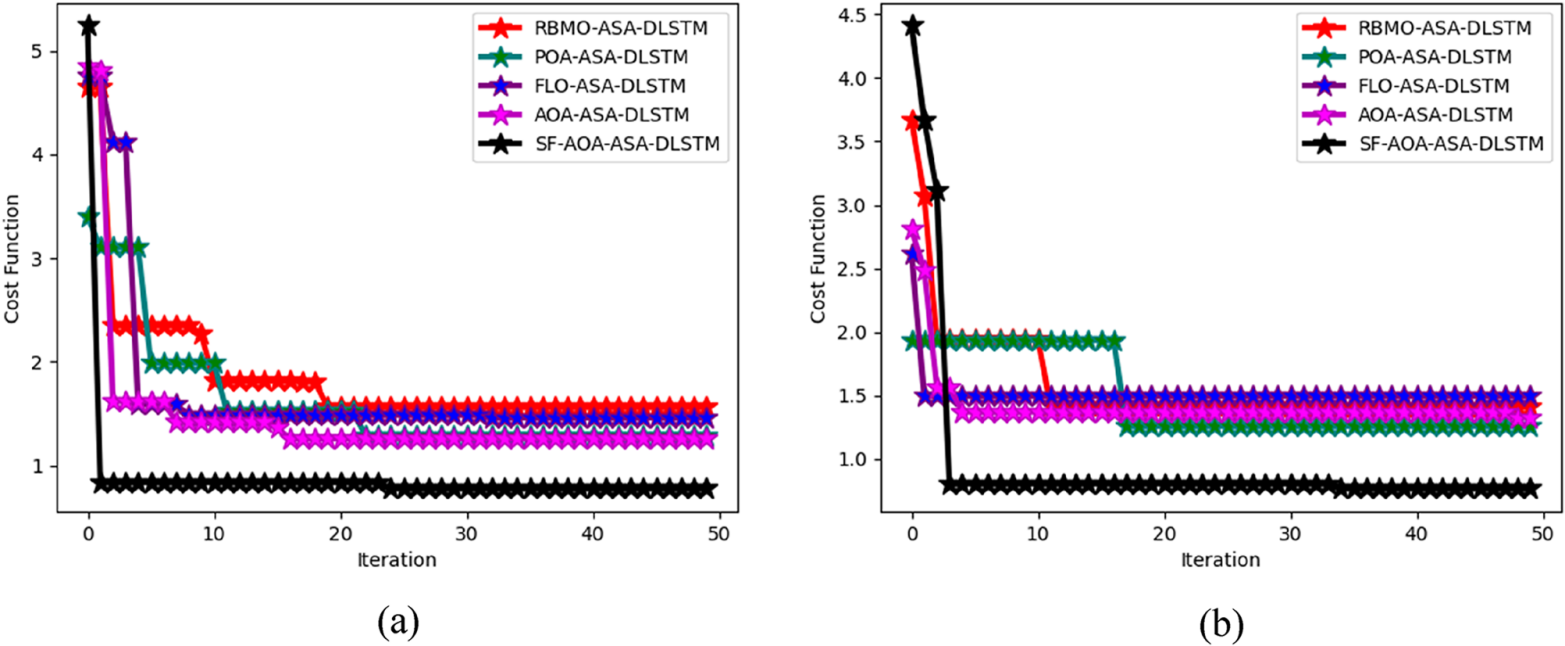
Convergences analysis of the proposed blockchain-based secure IoT communication regarding “(**a**) Dataset 1, and (**b**) Dataset 2”.

Figure 7 provides the convergence analysis of the proposed blockchain-based secured IoT communication framework. Convergence analysis ensures that the proposed SF-AOA-ASA-DLSTM system reaches stability during its learning phases. This implies that the model’s parameters settle into consistent values, providing dependable and predictable performance for secure key generation and data exchange. By validating convergence, it confirms that the SF-AOA-ASA-DLSTM network has successfully identified and prioritized key features necessary for optimal key generation in EGC, thereby improving both the security robustness and the effectiveness of encryption and decryption operations. Furthermore, this convergence indicates that the SF-AOA-ASA-DLSTM framework efficiently scales to support numerous IoT devices and complex data flows, maintaining consistent performance as the network expands. Thus, the convergence analysis is essential for verifying that the combined approach is reliable, efficient, and suitable for practical use in IoT scenarios.

### Accuracy analysis of the proposed blockchain-based secure IoT communication

**Fig. 8:**
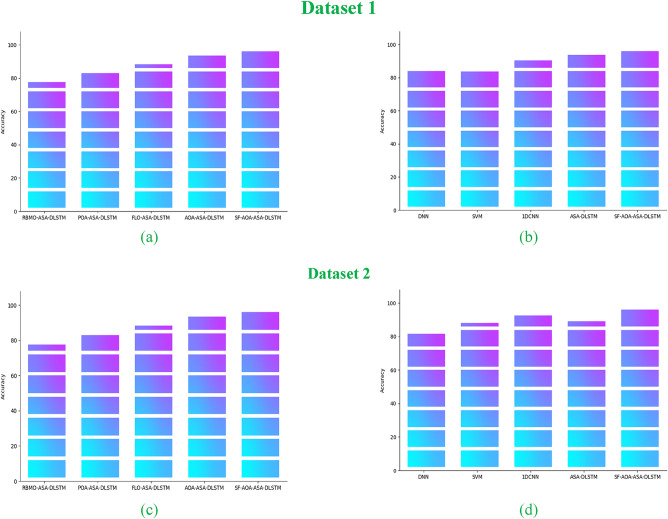
Accuracy analysis of the proposed blockchain-based Secure IoT communication in terms of “(**a**) Algorithms and (**b**) Techniques”.

Accuracy analysis of the proposed blockchain-based secure IoT communication is visually represented in Fig. 8. Evaluating accuracy is essential for confirming the effectiveness of the security measures within the blockchain structure, ensuring that IoT devices communicate safely and withstand potential threats, including data manipulation and unauthorized entry. By confirming the precision of the communications, blockchain technology protects the integrity of the data shared among IoT devices. The accuracy of the proposed SF-AOA-ASA-DLSTM network is 95.3%, which is better than RBMO-ASA-DLSTM, POA-ASA-DLSTM, FLO-ASA-DLSTM, and AOA-ASA-DLSTM using dataset 1. If the proposed scheme achieves high accuracy and verifies the transactions logged on the blockchain, then the transaction process ensures that each transaction is correctly documented and accurately represents the interactions between IoT devices, thus boosting user confidence.

### Performance analysis of the proposed blockchain-based secure IoT communication

**Fig. 9:**
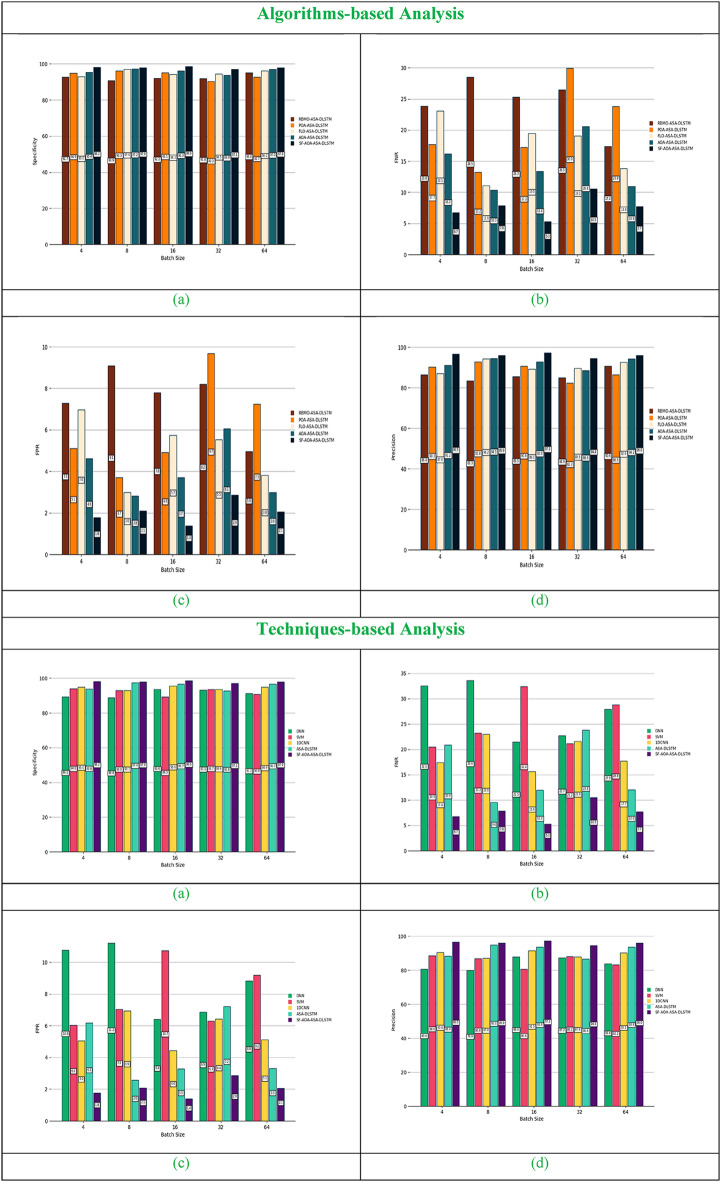
Performance analysis of the proposed blockchain-based secure IoT communication over conventional Algorithms and techniques using Dataset 1 in terms of “(**a**) Specificity, (**b**) FNR, (**c**) FPR, and (**d**) Precision”.

**Fig. 10:**
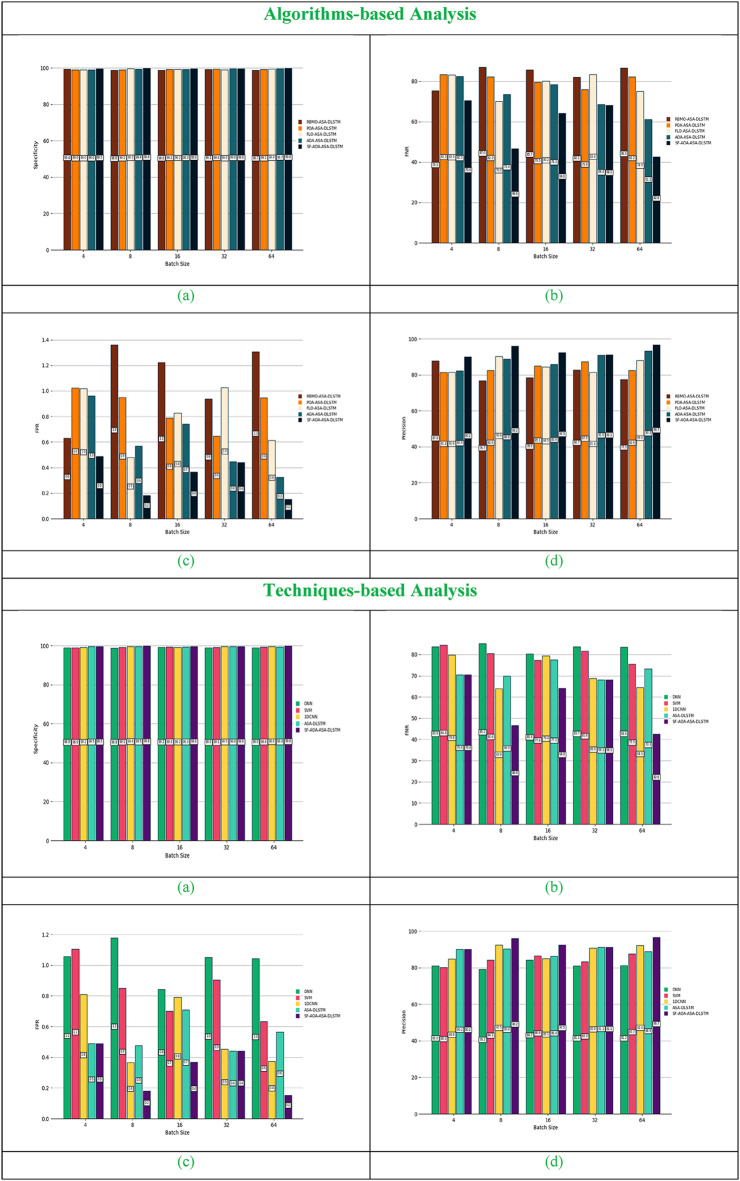
Performance analysis of the proposed blockchain-based secure IoT communication over conventional Algorithms and techniques using Dataset 2 in terms of “(**a**) Specificity, (**b**) FNR, (**c**) FPR, and (**d**) Precision”.

The evaluation of the proposed blockchain-based secure IoT communication’s performance is conducted by varying distinct blockchain parameters, which greatly impact the system’s effectiveness and security level. The graphical views of these performance analyses using both datasets are provided in Figs. 9 and 10. Increasing the block size allows more transactions in each block, which improves the throughput of the proposed SF-AOA-ASA-DLSTM network. The specificity of the proposed SF-AOA-ASA-DLSTM network is 97.1%, which is better than RBMO-ASA-DLSTM, POA-ASA-DLSTM, FLO-ASA-DLSTM, and AOA-ASA-DLSTM. High specificity reduces the delay that leads to more frequent block generation and greater consensus overhead. Conducting performance assessments by adjusting factors such as blockchain type, block dimensions, consensus protocols, and transaction volume helps to analyze their effects on communication delay, throughput, and overall security in IoT networks. Thus, the proposed SF-AOA-ASA-DLSTM model achieved an optimal outcome that delivers secure communication in IoT environments.

### Encryption and decryption time analysis of the proposed model

**Fig. 11:**
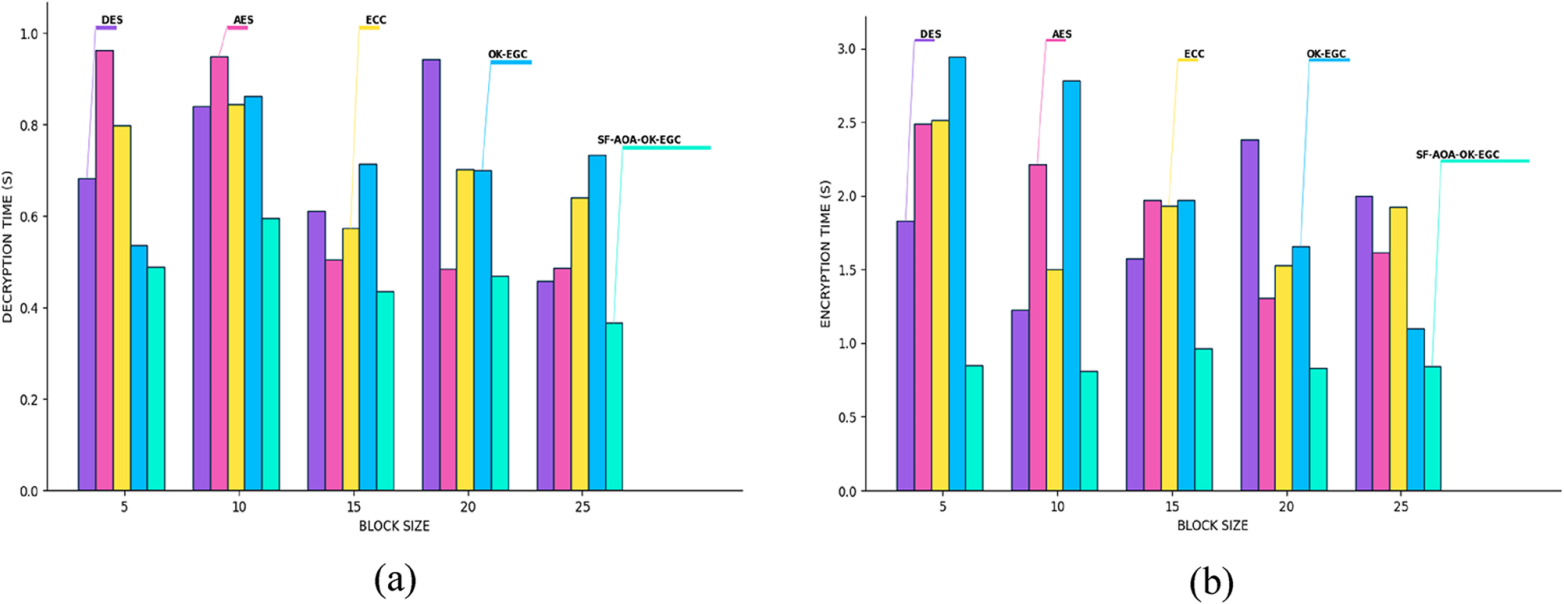
Analysis of the proposed cryptography model in terms of “(**a**) Decryption time and (**b**) Encryption time”.

Figure 11 provides the encryption and decryption time analysis of the proposed framework. Evaluating the time taken for encryption and decryption with the support of cryptographic methods ensures that security tasks do not cause significant delays in data transfer or burden device resources. The encryption and decryption time of the proposed model SF-AOA-OK-EGC is decreased, which minimizes the computational resources needed, which is especially beneficial for resource-limited devices. Efficient encryption and decryption allow the system to handle a larger volume of data and more users without degradation in performance. With faster encryption and decryption processes, the SF-AOA-OK-EGC model manages high amounts of data and accommodates more users or devices at the same time without compromising system performance. As a result, the proposed scheme delivers strong security protections while allowing efficient data transmission and real-time communication. Therefore, SF-AOA-OK-EGC achieves a balance between maintaining security and ensuring operational effectiveness, preventing security mechanisms from creating delays, and making the framework well-suited for real-world applications in challenging conditions.

### Performance analysis of proposed transmission model by varying batch sizes

The numerical validations of the designed model are provided in Tables 2 and 3 for two datasets. This evaluation verifies that data transmitted between IoT devices remains intact and genuine, guaranteeing the proper performance of blockchain and validation features. By examining various measures, the SF-AOA-ASA-DLSTM system confirms that messages are delivered and understood correctly, reducing errors that may interfere with IoT processes. The accuracy of the proposed model is 96.5%, and the FNR of the designed model is 1.77% at batch size 4. High accuracy indicates that the SF-AOA-ASA-DLSTM model effectively makes correct predictions, ensuring dependable and consistent outcomes in real-world scenarios. Consequently, the performance assessment plays a vital role in ensuring that secure data transfers in blockchain-based IoT networks uphold data integrity, dependability, and confidence, contributing to robust and efficient IoT communication.

**Table 2:** Performance analysis of the proposed model by varying batch size values for the first dataset.

Algorithms-based Analysis					
Batch sizes	RBMO-ASA-DLSTM [27]	POA-ASA-DLSTM [28]	FLO-ASA-DLSTM [29]	AOA-ASA-DLSTM [26]	SF-AOA-ASA-DLSTM
**Accuracy**					
4	86.44731	90.302028	86.946649	91.1772487	96.510141
8	83.376323	92.889109	94.161155	94.5392416	95.919312
16	85.529101	90.591931	89.188713	92.8439153	97.272928
32	84.766314	82.368827	89.481922	88.5295414	94.444444
64	90.512566	86.491402	92.595899	94.2184744	95.978836
**Sensitivity**					
4	76.150506	82.319426	76.896088	83.7974222	93.254729
8	71.513724	86.755663	88.950311	89.6580928	92.166645
16	74.718322	82.7682	80.51374	86.6391015	94.69736
32	73.510882	70.036853	80.942415	79.3889284	89.473684
64	82.64373	76.205741	86.179212	89.080334	92.279318
**Specificity**					
4	92.710383	94.903385	93.02877	95.3756226	98.224623
8	90.908108	96.290204	97.003458	97.1838885	97.912307
16	92.198695	95.090363	94.262753	96.2923583	98.613812
32	91.810036	90.314103	94.468694	93.9430637	97.142857
64	95.040375	92.74887	96.179416	97.015713	97.941693
**Precision**					
4	86.402116	90.300926	86.970899	91.1574074	96.511243
8	83.316799	92.840608	94.183201	94.5205026	95.909392
16	85.525794	90.648148	89.140212	92.8505291	97.265212
32	84.887566	82.328042	89.52381	88.587963	94.444444
64	90.555556	86.474868	92.645503	94.2030423	95.965608
**FPR**					
4	7.2896169	5.096615	6.9712304	4.62437742	1.775377
8	9.0918921	3.7097963	2.9965418	2.81611149	2.0876932
16	7.8013047	4.909637	5.7372467	3.70764165	1.3861884
32	8.1899642	9.6858971	5.5313057	6.05693625	2.8571429
64	4.9596249	7.2511302	3.8205837	2.98428695	2.0583075
**FNR**					
4	23.849494	17.680574	23.103912	16.2025778	6.745271
8	28.486276	13.244337	11.049689	10.3419072	7.8333545
16	25.281678	17.2318	19.48626	13.3608985	5.3026401
32	26.489118	29.963147	19.057585	20.6110716	10.526316
64	17.35627	23.794259	13.820788	10.919666	7.7206818
**FDR**					
4	80.953045	86.125654	81.623786	87.3226052	94.855044
8	76.965374	89.695053	91.491993	92.0251127	94.000778
16	79.757609	86.529143	84.607659	89.6373388	95.964111
32	78.790669	75.686686	85.017115	83.7365591	91.891892
64	86.418935	81.016188	89.295447	91.5700992	94.08637
**F1 score**					
4	0.7083807	0.7889505	0.7188759	0.8074324	0.9224687
8	0.6453654	0.8438905	0.8713543	0.87948498	0.9095208
16	0.6895573	0.7951759	0.7654395	0.84300007	0.9392457
32	0.6742325	0.6249599	0.7717727	0.75186689	0.8774536
64	0.7934756	0.709361	0.8377543	0.87254842	0.9108193
**MCC**					
4	13.530093	9.6974206	13.065476	8.81283069	3.4904101
8	16.593915	7.0866402	5.8498677	5.45138889	4.0757275
16	14.469246	9.4361772	10.787037	7.15939153	2.7232143
32	15.294312	17.61078	10.539021	11.4996693	5.5555556
64	9.5089286	13.500331	7.4289021	5.77380952	4.0145503
**Techniques-based Analysis**					
Batch sizes	DNN [31]	SVM [32]	1DCNN [33]	ASA-DLSTM [34][35]	SF-AOA-ASA-DLSTM
**Accuracy**					
4	80.57209	88.587963	90.454145	88.3575838	96.510141
8	79.825838	86.872795	87.003968	94.9713404	95.919312
16	87.96627	80.670194	91.511243	93.6232363	97.272928
32	87.179233	88.154762	87.915564	86.5002205	94.444444
64	83.77425	83.193342	90.265653	93.6067019	95.978836
**Sensitivity**					
4	67.460208	79.501558	82.604097	79.1353272	93.254729
8	66.416392	76.803626	76.983152	90.4327014	92.166645
16	78.514299	67.624861	84.367662	88.0078331	94.69736
32	77.273926	78.81852	78.438026	76.1868724	89.473684
64	72.066769	71.228726	82.276496	87.9809784	92.279318
**Specificity**					
4	89.2481	93.959484	94.962343	93.8261633	98.224623
8	88.79507	92.964175	93.064105	97.4154598	97.912307
16	93.600999	89.268471	95.554903	96.7087397	98.613812
32	93.149015	93.704524	93.568198	92.7863036	97.142857
64	91.185153	90.81885	94.869255	96.6983227	97.941693
**Precision**					
4	80.588624	88.611111	90.400132	88.3730159	96.511243
8	79.857804	86.848545	87.03373	94.9603175	95.909392
16	87.972884	80.595238	91.484788	93.6276455	97.265212
32	87.175926	88.154762	87.91336	86.5542328	94.444444
64	83.806217	83.17791	90.234788	93.6078042	95.965608
**FPR**					
4	10.7519	6.0405157	5.0376566	6.1738367	1.775377
8	11.20493	7.0358249	6.9358947	2.58454024	2.0876932
16	6.3990007	10.731529	4.4450966	3.29126031	1.3861884
32	6.8509849	6.2954761	6.4318018	7.21369644	2.8571429
64	8.8148468	9.1811504	5.1307445	3.30167731	2.0583075
**FNR**					
4	32.539792	20.498442	17.395903	20.8646728	6.745271
8	33.583608	23.196374	23.016848	9.56729861	7.8333545
16	21.485701	32.375139	15.632338	11.9921669	5.3026401
32	22.726074	21.18148	21.561974	23.8131276	10.526316
64	27.933231	28.771274	17.723504	12.0190216	7.7206818
**FDR**					
4	73.442327	83.809524	86.32646	83.4994532	94.855044
8	72.51952	81.517809	81.700503	92.6412233	94.000778
16	82.974908	73.542547	87.782202	90.7308007	95.964111
32	81.926812	83.225625	82.905836	81.0403282	91.891892
64	77.494419	76.740958	86.072076	90.7072131	94.08637
**F1 score**					
4	0.5888847	0.7530122	0.7920652	0.74818553	0.9224687
8	0.5739633	0.7172323	0.7200761	0.88886528	0.9095208
16	0.7400092	0.5905698	0.8145129	0.85973304	0.9392457
32	0.7236315	0.743922	0.738934	0.70971367	0.8774536
64	0.6537265	0.6417674	0.788128	0.85937284	0.9108193
**MCC**					
4	19.436177	11.423611	9.5188492	11.6501323	3.4904101
8	20.190146	13.115079	13.010913	5.02314815	4.0757275
16	12.037037	19.292328	8.4755291	6.37896825	2.7232143
32	12.819114	11.845238	12.083333	13.5267857	5.5555556
64	16.241733	16.798942	9.7189153	6.39384921	4.0145503

**Table 3:** Performance analysis of the proposed model, varying batch size values for the second dataset.

Algorithms-based Analysis					
Batch sizes	RBMO-ASA-DLSTM [27]	POA-ASA-DLSTM [28]	FLO-ASA-DLSTM [29]	AOA-ASA-DLSTM [26]	SF-AOA-ASA-DLSTM
**Accuracy**					
4	87.74068292	81.48433526	81.57093622	82.39593363	90.22694029
8	76.66698057	82.56147876	90.3989541	88.85250201	96.1794365
16	78.59323597	85.02049732	84.44777803	85.88954329	92.51038067
32	82.75628693	87.46996689	81.40917548	91.03398384	91.16291719
64	77.40898538	82.63602248	88.06182816	93.34048261	96.74287082
**Sensitivity**					
4	24.5485535	16.66255941	16.74858419	17.54076705	29.56155009
8	13.00330146	17.71163243	29.97231869	26.59395922	53.36482297
16	14.29997514	20.52056693	19.79930968	21.67608435	35.95481746
32	17.90712649	24.0887732	16.59886775	31.57475228	31.91824234
64	13.4830273	17.78444327	25.10650703	38.91676125	57.44861161
**Specificity**					
4	99.36961195	98.97502636	98.98320621	99.03740932	99.51067643
8	98.63947149	99.05001758	99.52007058	99.43266121	99.8190928
16	98.7763848	99.21007479	99.17118102	99.2603356	99.63246715
32	99.0614093	99.35344805	98.97207505	99.55320174	99.55978477
64	98.6938402	99.05377215	99.38625239	99.67703251	99.84706416
**Precision**					
4	87.75454435	81.43496215	81.56552366	82.38128011	90.23926157
8	76.73677179	82.57965265	90.40929517	88.8466054	96.16513501
16	78.58082669	85.10789037	84.47330068	85.9195984	92.49220679
32	82.74968625	87.47722764	81.39852638	91.01149751	91.13193798
64	77.46245091	82.6332942	88.03590948	93.34642322	96.74001053
**FPR**					
4	0.630388049	1.024973639	1.016793789	0.962590678	0.489323573
8	1.360528514	0.949982421	0.479929419	0.56733879	0.1809072
16	1.223615201	0.789925206	0.828818978	0.739664397	0.367532848
32	0.938590704	0.646551949	1.02792495	0.446798256	0.440215234
64	1.306159798	0.946227855	0.613747614	0.322967489	0.152935836
**FNR**					
4	75.4514465	83.33744059	83.25141581	82.45923295	70.43844991
8	86.99669854	82.28836757	70.02768131	73.40604078	46.63517703
16	85.70002486	79.47943307	80.20069032	78.32391565	64.04518254
32	82.09287351	75.9112268	83.40113225	68.42524772	68.08175766
64	86.5169727	82.21555673	74.89349297	61.08323875	42.55138839
**FDR**					
4	87.74005285	81.48657949	81.57118225	82.3965997	90.22638023
8	76.66380824	82.56065268	90.39848405	88.85277004	96.18008656
16	78.59380003	85.01652491	84.44661791	85.88817714	92.51120676
32	82.75658696	87.46963686	81.40965953	91.03500594	91.16432533
64	77.40655513	82.63614649	88.06300628	93.34021258	96.74300084
**F1 score**					
4	0.424934379	0.313678339	0.315159233	0.327704	0.483664694
8	0.249345308	0.330432898	0.488181778	0.44969458	0.700805286
16	0.273429332	0.371967702	0.361586044	0.387736733	0.550003697
32	0.333398492	0.419156412	0.312727145	0.505365193	0.508971906
64	0.258482614	0.331514842	0.431725889	0.578408692	0.731858751
**MCC**					
4	12.25994715	18.51342051	18.42881775	17.6034003	9.773619765
8	23.33619176	17.43934732	9.601515949	11.14722996	3.819913437
16	21.40619997	14.98347509	15.55338209	14.11182286	7.48879324
32	17.24341304	12.53036314	18.59034047	8.964994056	8.835674669
64	22.59344487	17.36385351	11.93699372	6.659787421	3.256999165
**Techniques-based Analysis**					
Batch sizes	DNN [31]	SVM [32]	1DCNN [33]	ASA-DLSTM [34][35]	SF-AOA-ASA-DLSTM
**Accuracy**					
4	80.99575268	80.32103083	84.77486389	90.22694029	90.22694029
8	79.23354626	84.10850291	92.54325208	90.44401476	96.1794365
16	84.27105574	86.55652033	85.11770338	86.47233962	92.51038067
32	81.06880024	83.24165718	90.8693628	91.16291719	91.16291719
64	81.20063789	87.70209092	92.3579929	88.89078597	96.74287082
**Sensitivity**					
4	16.22761121	15.64132757	20.20160863	29.56155009	29.56155009
8	14.77922353	19.3956335	36.06497882	30.08075076	53.36482297
16	19.58058408	22.64379682	20.62746157	22.50697833	35.95481746
32	16.2949071	18.42414037	31.14955965	31.91824234	31.91824234
64	16.40911713	24.48097497	35.45684845	26.67047263	57.44861161
**Specificity**					
4	98.94429995	98.89537686	99.19175466	99.51067643	99.51067643
8	98.8224804	99.14974204	99.63447142	99.52222456	99.8190928
16	99.15750819	99.30025935	99.20942725	99.29137506	99.63246715
32	98.95028598	99.09502362	99.54631567	99.55978477	99.55978477
64	98.9577575	99.36690485	99.62543174	99.43515385	99.84706416
**Precision**					
4	80.98761184	80.26193272	84.80324683	90.23926157	90.23926157
8	79.22958585	84.13222137	92.53066677	90.44775515	96.16513501
16	84.24760131	86.58151492	85.07752723	86.42261447	92.49220679
32	81.07971337	83.27598073	90.88903283	91.13193798	91.13193798
64	81.18497227	87.70697543	92.36063317	88.89113801	96.74001053
**FPR**					
4	1.05570005	1.104623138	0.808245341	0.489323573	0.489323573
8	1.1775196	0.850257957	0.365528575	0.477775444	0.1809072
16	0.842491814	0.699740645	0.790572752	0.70862494	0.367532848
32	1.049714016	0.904976384	0.453684333	0.440215234	0.440215234
64	1.042242498	0.633095145	0.374568264	0.564846149	0.152935836
**FNR**					
4	83.77238879	84.35867243	79.79839137	70.43844991	70.43844991
8	85.22077647	80.6043665	63.93502118	69.91924924	46.63517703
16	80.41941592	77.35620318	79.37253843	77.49302167	64.04518254
32	83.7050929	81.57585963	68.85044035	68.08175766	68.08175766
64	83.59088287	75.51902503	64.54315155	73.32952737	42.55138839
**FDR**					
4	80.99612272	80.32371711	84.77357376	90.22638023	90.22638023
8	79.23372628	84.1074248	92.54382414	90.44384475	96.18008656
16	84.27212185	86.55538421	85.11952957	86.47459985	92.51120676
32	81.06830419	83.24009701	90.8684687	91.16432533	91.16432533
64	81.20134996	87.7018689	92.35787289	88.89076997	96.74300084
**F1 score**					
4	0.30666133	0.296768543	0.367332078	0.483664694	0.483664694
8	0.281993025	0.355742866	0.551100041	0.489349776	0.700805286
16	0.358319535	0.400614556	0.373160985	0.39862755	0.550003697
32	0.307808143	0.341365797	0.500960884	0.508971906	0.508971906
64	0.309626031	0.424068515	0.545171383	0.450615738	0.731858751
**MCC**					
4	19.00387728	19.67628289	15.22642624	9.773619765	9.773619765
8	20.76627372	15.8925752	7.456175863	9.556155253	3.819913437
16	15.72787815	13.44461579	14.88047043	13.52540015	7.48879324
32	18.93169581	16.75990299	9.131531296	8.835674669	8.835674669
64	18.79865004	12.2981311	7.642127113	11.10923003	3.256999165

### Statistical analysis of the proposed method

Statistical evaluation provides a valuable understanding of how effectively the data is conveyed securely throughout the network. The mean value of the proposed model is 0.86%, which is lower than that of existing models. Additionally, analyzing computational load and bandwidth usage statistics supports the development of resource allocation strategies, helping the IoT system to run smoothly while upholding security standards. Statistical analysis ensures that secure data transmission using the SF-AOA-ASA-DLSTM system improves the quality of service demands in IoT applications. Therefore, statistical analysis is an essential method to assess the security, performance, and dependability of data communication in blockchain-enabled IoT environments. Table 4 provides the statistical analysis of the designed technique. From Table 4, the proposed SF-AOA-ASA-DLSTM model attains the median, best-case error values and lowest mean across both datasets, which illustrating optimal performance. Although, the worst-case value is higher while comparing other baseline approaches. This modification happened because of the stochastic exploration ability of the SF-AOA optimizer, which establishes variability across runs. It is helpful for preventing premature convergence and utilized to discover better global optima. Accordingly, the median values are lower than the other baseline approaches, which denote that the majority of runs yield high performance.

**Table 4:** Statistical analysis of the designed model.

Metrics	RBMO-ASA-DLSTM [27]	POA-ASA-DLSTM [28]	FLO-ASA-DLSTM [29]	AOA-ASA-DLSTM [26]	SF-AOA-ASA-DLSTM
Dataset 1					
Standard deviation	0.634578	0.565445	0.805877	0.696411	0.621662
Median	1.561426	1.289602	1.486701	1.260006	0.77865
Mean	1.854927	1.613594	1.722574	1.467731	0.896236
Best	1.561426	1.289602	1.46326	1.260006	0.77865
Worst	4.644347	3.395123	4.758177	4.854323	5.242665
**Dataset 2**					
Standard deviation	0.422071	0.320109	0.157004	0.254333	0.710953
Worst	3.673265	1.932076	2.614255	2.806459	4.413894
Best	1.405852	1.256326	1.492797	1.326038	0.762387
Median	1.41526	1.256326	1.492797	1.369136	0.802269
Mean	1.584776	1.486081	1.515226	1.426303	0.965179

### Statistical significance test

Table 5 provides the statistical significance test over existing algorithms. The Friedman Aligned Ranks test is a non-parametric statistical examination employed to estimate whether there are significant functionality variations between distinct algorithms across some experimental conditions. From this validation, it is displayed that the designed SF-AOA-ASA-DLSTM is statistically significant.

**Table 5:** Statistical significance test.

Friedman Aligned Ranks test (significance level of 0.005)			
Comparison	Statistic	Adjusted *p*-value	Result
RBMO-ASA-DLSTM [27] vs. Proposed SF-AOA-ASA-DLSTM	1.78885	0.73638	H0 is accepted
FLO-ASA-DLSTM [29] vs. RBMO-ASA-DLSTM [27]	1.34164	1	
POA-ASA-DLSTM [28] vs. Proposed SF-AOA-ASA-DLSTM	1.34164	1	
FLO-ASA-DLSTM [29] vs. POA-ASA-DLSTM [28]	0.89443	1	
RBMO-ASA-DLSTM [27] vs. AOA-ASA-DLSTM [26]	0.89443	1	
Proposed SF-AOA-ASA-DLSTM vs. AOA-ASA-DLSTM [26]	0.89443	1	
FLO-ASA-DLSTM [29] vs. Proposed SF-AOA-ASA-DLSTM	0.44721	1	
POA-ASA-DLSTM [28] vs. RBMO-ASA-DLSTM [27]	0.44721	1	
POA-ASA-DLSTM [28] vs. AOA-ASA-DLSTM [26]	0.44721	1	
FLO-ASA-DLSTM [29] vs. AOA-ASA-DLSTM [26]	0.44721	1	

### Ablation study

Table 6 provides the ablation study of the suggested SF-AOA-ASA-DLSTM. This study is utilized to understand the significance of individual components of the designed SF-AOA-ASA-DLSTM. In this study, it is shown that the designed SF-AOA-ASA-DLSTM provides superior performance to the existing methods in terms of accuracy, latency, and training time. Hence, it is confirmed that the designed SF-AOA-ASA-DLSTM is effective.

**Table 6:** Ablation study of the proposed model.

Model Variant	Accuracy (%)	Latency (ms)	Training Time (s)
Baseline LSTM	93.84	28.7	182
LSTM + Sparse Attention	95.96	24.3	169
Dense LSTM (without optimization)	96.72	23.1	158
SA-DLSTM (without SF-AOA tuning)	97.88	21.9	151
ASA-DLSTM + SF-AOA (without OK-EGC)	98.43	21.1	143
Proposed ASA-DLSTM + SF-AOA + OK-EGC + Blockchain	98.43	21.4	146

### Confusion matrix analysis

**Fig. 12:**
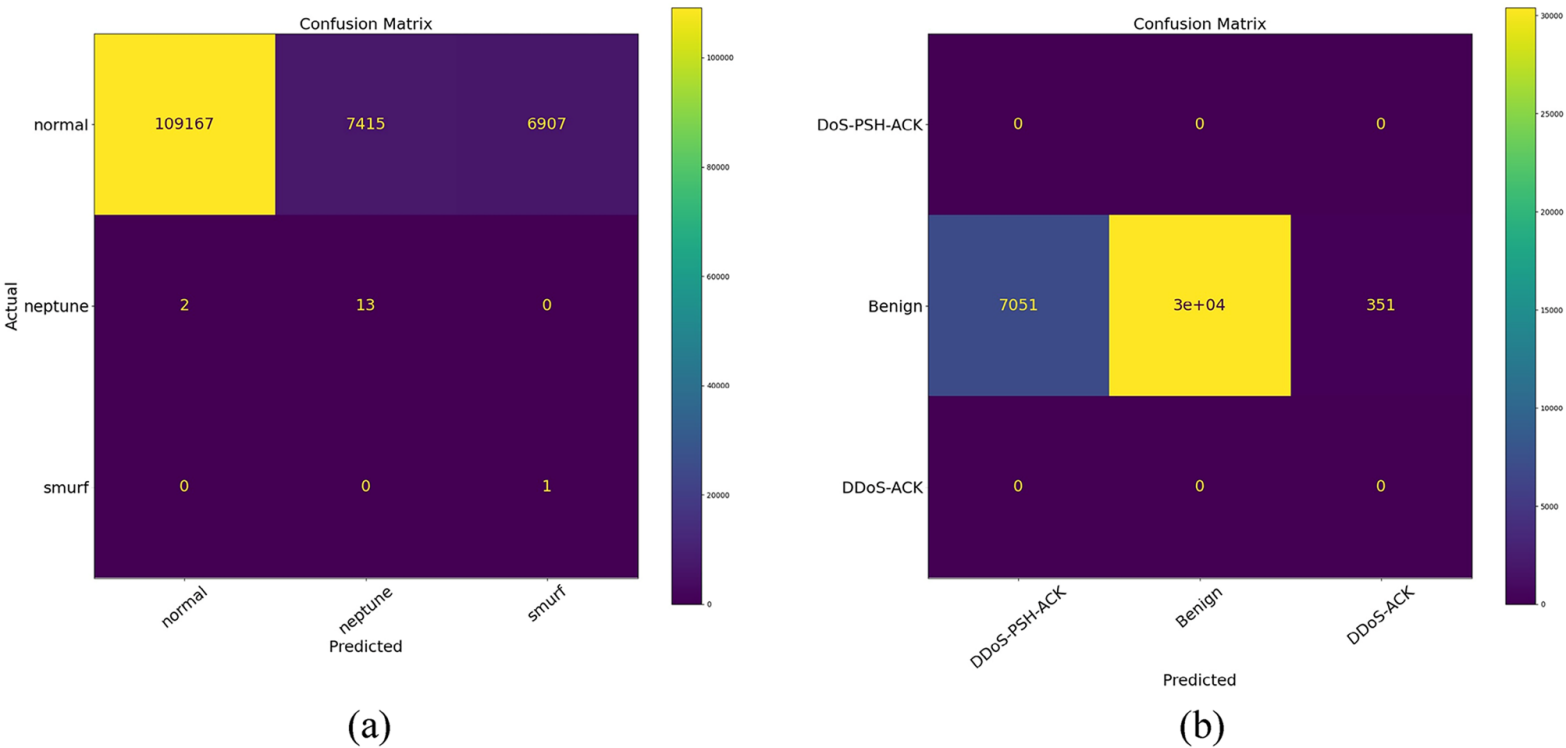
Confusion matrix analysis of the proposed technique for Datasets “(**a**) 1 and (**b**) 2”.

The confusion matrix validation of the designed ASA-DLSTM is provided in Fig. 12. It is an effective validation for the detection techniques, connecting forecasted over original classes to recognize the correct classifications. This analysis is done for two datasets, and the matrices elucidate that the implemented ASA-DLSTM provides more accurate detection results in both datasets. Therefore, the designed ASA-DLSTM-aided intrusion identification process is innovative and effective.

### Quantitative analysis of the computational overhead

The quantitative validation of the computational overhead, such as CPU cycles and memory usage during training vs. inference for the designed blockchain-based secure data transmission approach, is shown in Tables 7 and 8. In addition, a comparison of the model’s complexity in terms of FLOPs with other baseline models is shown in Table 9. The given table results illustrate that the developed model performed with robust computational overheads. Thus, it is revealed that the updated SF-AOA algorithm tunes the network’s complexity without heavy cycle cost, and also efficiently manages the trade-off between computational complexity and performance.

**Table 7:** Validation of the computational overhead in terms of CPU cycles.

Model	Training CPU Cycles (×10⁹)	Inference CPU Cycles (×10⁶)
DNN [31]	8.5	12.3
1D-CNN [32]	10.2	15.6
LSTM [33]	12.8	18.4
ASA-DLSTM [34][35]	14.5	19.7
Proposed SF-AOA-ASA-DLSTM	13.9	17.2

**Table 8:** Validation of memory usage during training vs. inference.

Model	Training Memory	Inference Memory
DNN [31]	850 MB	210 MB
1D-CNN [32]	1020 MB	260 MB
LSTM [33]	1180 MB	310 MB
ASA-DLSTM [34][35]	1320 MB	340 MB
Proposed SF-AOA-ASA-DLSTM	1285 MB	295 MB

**Table 9:** Validation of the model’s complexity in terms of FLOPs.

Model	Parameters (M)	FLOPs (GFLOPs)	Model Size (MB)
DNN [31]	0.15	0.02	2.1
1D-CNN [32]	2.8	1.2	22.5
LSTM [33]	3.6	2.5	28.4
ASA-DLSTM [34][35]	4.2	3.1	32.7
Proposed SF-AOA-ASA-DLSTM	5	3.6	36.8

### Performance benefits of a designed approach

The experimental validation illustrates that the designed SF-AOA-ASA-DLSTM with OK-EGC and Blockchain mechanism offers more performance benefits across distinct validations, which are described below.

#### Convergence stability

Figure 7 guarantees that the designed SF-AOA-ASA-DLSTM technique achieves stable and rapid convergence for both data sources. The results elucidate that the learning process of the model efficiently attains an optimal solution without divergence. The convergence stability confirms that the optimized parameters remain consistent. It is highly important for effective key generation and encryption operations in complex IoT scenarios.

#### Improved detection performance

Figure 8 displays that the designed framework attains a maximum accuracy of 95.3%. Thus, the designed SF-AOA-ASA-DLSTM approach surpasses the conventional RBMO-ASA-DLSTM, POA-ASA-DLSTM, FLO-ASA-DLSTM, and AOA-ASA-DLSTM. The high accuracy value elucidates that the designed SF-AOA-ASA-DLSTM can precisely differentiate between the malicious and authorized traffic. Thus, the implemented SF-AOA-ASA-DLSTM improves the trust in the blockchain-aided IoT communication.

#### Outperformed classification measures

Figures 9 and 10 display that the suggested SF-AOA-ASA-DLSTM attains high specificity, low FPR, high precision, low FNR, and high F1 score rates. These outcomes explained that the designed SF-AOA-ASA-DLSTM gives a highly consistent intrusion identification performance compared to the existing algorithms and deep techniques.

#### Effective encryption and decryption

Figure 11 discloses that the introduced SF-AOA-OK-EGC technique needs very little computational time than other models. It makes the designed SF-AOA-OK-EGC highly suitable for resource-limited IoT systems. The faster cryptographic task guarantees lower energy usage and minimized processing delay.

#### Effective batch size validation

Tables 2 and 3 describe that the designed framework attains low FNR values and high accuracy even with high batch sizes. This illustrates the high robustness of the designed framework. In addition, these validations guarantee that the designed framework can handle the huge amount of IoT data without compromising the performance rate.

#### Statistical superiority

Tables 4 and 5 elucidate that the designed model has a stronger statistical significance than the other algorithms. It also ensures the designed SF-AOA-ASA-DLSTM’s stability and robustness across data sources.

#### Ablation study

Table 6 confirms that the SA in the suggested ASA-DLSTM + SF-AOA + OK-EGC + Blockchain minimizes the training time and latency. Also, the SF-AOA enhances the convergence and accuracy rate. In addition, the OK-EGC and Blockchain improve the security of the framework without facing overhead issues.

#### Confusion matrix validation

Figure 12 gives the designed ASA-DLSTM technique’s confusion matrix validation, which elucidates that the implemented ASA-DLSTM can reduce the false alarms in the intrusion identification tasks.

Thus, the designed framework achieves superior performance by guaranteeing quick convergence, effective cryptographic tasks, high detection accuracy, and low computational burdens. These merits make the designed approach a relatively reliable solution for secure blockchain-based IoT communication.

### Cyberattack scenario evaluation

To validate the robustness of the designed SF-AOA-ASA-DLSTM with OK-EGC and blockchain model, some cyberattack scenarios are simulated in the controlled IoT scenario. The goal of this validation is to validate how efficiently the designed model identifies, eliminates, and recovers from distinct attack patterns normally encountered in the real-world IoT networks.

### Evaluation setup

The validation considers an IoT model including blockchain nodes, edge systems, and heterogeneous sensor nodes. Both malicious and benign traffic features are given to the network. The designed SF-AOA-ASA-DLSTM technique is trained to differentiate between abnormal and normal behaviors. At the same time, the implemented OK-EGC guarantees secure communication, and the blockchain ensures the maintenance of immutable logs.

### Cyberattack scenarios

*Scenario 1: False Data Injection Attack (FDIA)*.

An attacker includes forged sensor readings to change the system’s decisions. It results in inaccurate control actions and network instability. The designed SF-AOA-ASA-DLSTM detects the abnormal patterns. The SA separates the anomaly features, and the blockchain eliminates the fabricated data from being stored permanently. Hence, the designed SF-AOA-ASA-DLSTM with OK-EGC and blockchain model achieves high detection accuracy.

*Scenario 2: Man-in-the-Middle (MITM) Attack*.

The attacker changes the data at the transmission time among the servers and IoT nodes. Due to this, the data integrity and confidentiality are highly affected. The designed OK-EGC encrypts the data by employing the optimally selected elliptic Galois keys. This generation of dynamic key eliminates session hijacking. Therefore, the encrypted payloads become infeasible to decrypt.

*Scenario 3: Replay Attack*.

The previously valid packets are again transmitted to confuse the network. This creates deceptive updates for states. The suggested blockchain timestamps detect the old transactions, and the designed ASA-DLSTM flags the repeated traffic features. Hence, by utilizing the designed framework, the replay attacks are mitigated with high consistency.

### Practical deployment scenarios and threat models

To estimate the designed SF-AOA-ASA-DLSTM with OK-EGC and blockchain model’s real-world applicability, the practical deployment scenarios and the specific threat models resolved by the designed model are explained.

#### Practical deployment scenarios

The suggested model is implemented to work in different blockchain-based IoT scenarios. In these scenarios, scalable, intelligent, and secure communication is significant.

#### Smart healthcare systems

In these systems, continuously, the IoT sensors observe the patient’s vitals, like blood pressure, oxygen levels, and so on. The suggested approach allows real-time intrusion detection to eliminate tampering with the healthcare data. By utilizing the OK-EGC, the secure key generation and encryption process is performed to protect the sensitive patient data. In addition, the data traceability and integrity are guaranteed by the blockchain logging. Hence, these operations meet the privacy demands and mitigate the cyber-attacks.

#### Smart grid and energy management

In this application, numerous smart meters transmit data for load balancing and demand responses. The designed model identifies the abnormal traffic patterns generated by the false data injection. The blockchain is employed to handle the immutable transaction records. In addition, the suggested work performs the fast encryption process for real-time devices.

#### Smart manufacturing

In the industrial IoT, the sensors manage the production lines and robotic arms. The designed technique eliminates the command injection threats and guarantees the tamper-proof logs for audits. In addition, the designed model maintains low delay for the real-time automation.

**Threat models**: The suggested approach considers a strong adversarial technique, where the intruders can utilize distinct IoT environment layers. The intruder is considered to be a machine learning-aided attack, has access to the communication channels, and compromises the IoT system subsets. The network can face the FDIA, MITM, replay attacks, and so on. These attacks affect the system by inserting incorrect sensor data, altering messages, and reusing the old packets. To resolve these concerns, the designed ASA-DLSTM performs the anomaly detection, which prevents the incorrect data injection, the developed OK-EGC performs the encryption, which mitigates the data modification, and the suggested blockchain time stamps improve the security of the data packets. In this threat model, the following assumptions are made: (i) Most of the blockchain nodes are honest, (ii) lightweight cryptography is enough for the edge systems, (iii) trusted modal attribute initialization, and (iv) the adversary can’t destroy the elliptic curve complexity. Hence, the designed SF-AOA-ASA-DLSTM model works under a powerful adversarial design and offers multi-layer defense by integrating anomaly identification, optimized security, and better blockchain trust. It makes the designed framework applicable for the crucial IoT deployments.

#### Adversary capabilities

In this developed method, the adversary is designed with a full potential entity that can be designed with full rights to access the transmission channels in IoT platforms. It enables modification, replay, and interception of transmitted data, which can efficiently specify sophisticated attacks such as Man-In-The-Middle (MITM), replay attacks, and False Data Injection Attack (FDIA). It easily controls the system behaviour, and also this adversary technique leverages the machine learning strategies. These machine learning strategies are more helpful for preventing attacks and also supply moiré efficient anomaly detection mechanisms. In addition, this adversary can compromise a limited number of blockchain nodes, but it has not the ability to control the complicated attacks that ensure the integrity of the model. Especially, launching side-channel attacks such as timing and power is considered an upcoming work that is mitigated by the support of hardware or system design.

### Proposed framework performance in a real-time streaming data scenario

To display the practical applicability of the proposed framework in IoT platforms, a real-time streaming data scenario can be integrated with the static dataset validation. From this perspective, the given dataset is updated into time series segments to validate a continual data creation, where the received data are managed by a sliding window method. The developed ASA-DLSTM model works on each window to employ near real-time intrusion detection. It captures temporal dependencies, which efficiently reduces the computational overhead during the sparse attention model. In addition, the SF-AOA algorithm establishes the fine tuning of model parameters and optimization cryptographic keys with robust latency. The classified secure data is fed to the transmission process, which is carried out by the **OK**-EGC mechanism, which incorporates elliptic curve cryptography with Galois field functions to illustrate efficient data transmission in IoT devices. In addition, the key management strategy recommended detecting anomalies that enhance trustworthiness. At last, the effectiveness in this simulated streaming setting can be validated using metrics such as accuracy, inference time, and computational overhead. It is established that the suggested approach manages stable detection performance and minimum computational overhead. Thus, it is confirmed that the developed approach is suitable for the practical and time-sensitive IoT applications.

### Practical data flow

**Fig. 13:**
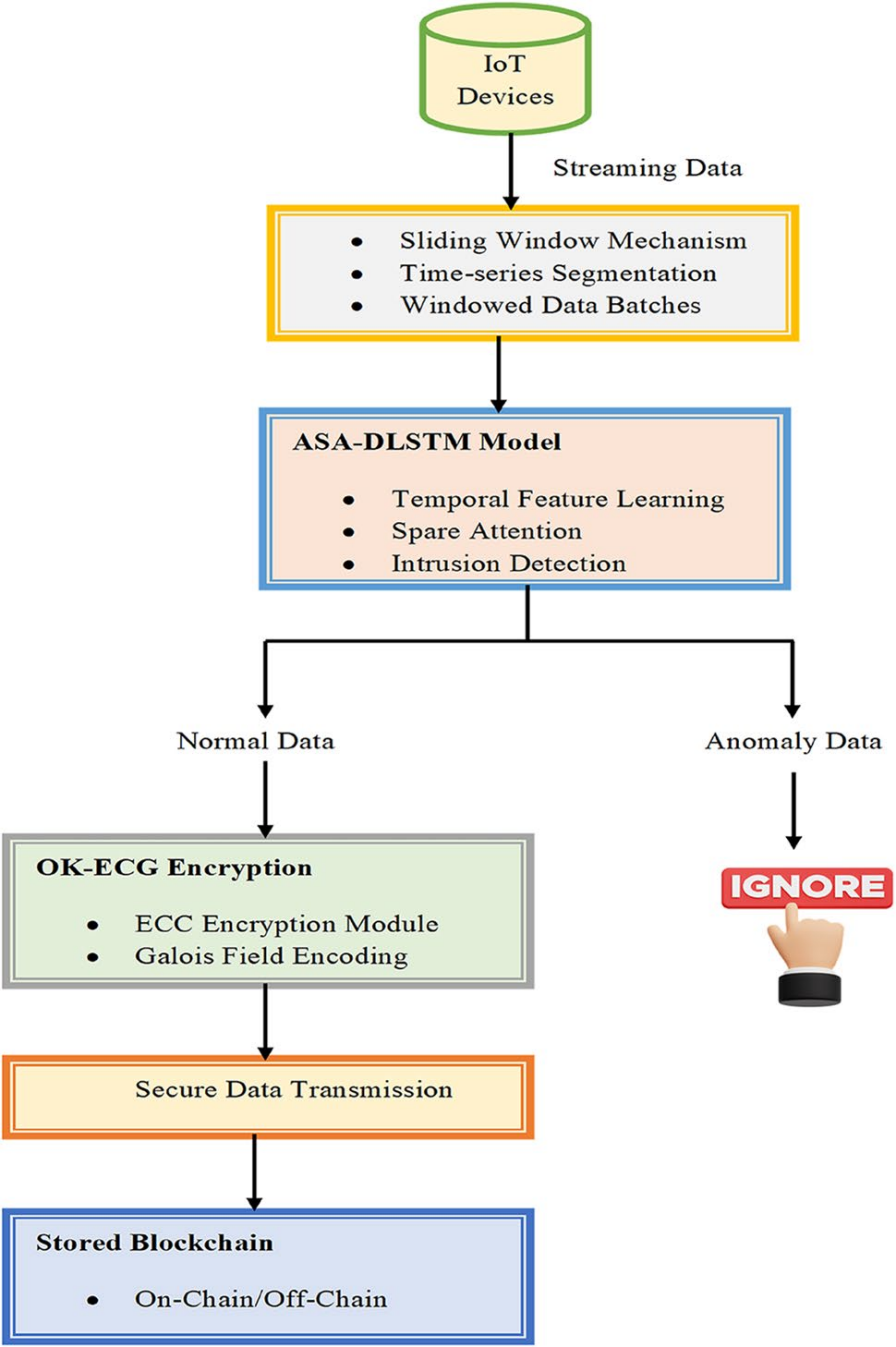
Data-flow diagram for blockchain storage.

In this developed framework, specific data stored on the blockchain, such as encrypted data, intrusion logs, key ID, raw data, processed data, hash, and keys and also the clear storage details on blockchain, is given in Table 10. In addition, the blockchain does not store encrypted IoT data or raw data straightforwardly owing to the scalability variables. Here, the encrypted data is stored off-chain, while the blockchain stores only the key identifiers, transaction metadata, cryptographic hash of the data, and intrusion detection logs. This on-chain/off-chain establishes robust traceability, low storage overhead, and data integrity. At this stage, the suggested ASA-DLSTM method allows intrusion-correlated metadata to the blockchain for auditability, while the OK-EGC mechanism ensures secure encryption. A detailed data-flow diagram for blockchain storage is visualized in Fig. 13.

**Table 10:** Specific data stored in the blockchain.

Stage	Data Type	Storage Location
Raw Data	Plaintext	Edge device
Processed Data	Clean/Attack	Edge
Encrypted Data	Ciphertext	Off-chain
Hash	SHA-256	Blockchain
Intrusion Logs	Metadata	Blockchain
Keys	Private	Secure vault
Key ID	Reference	Blockchain

## Conclusion

An efficient framework was developed for secure data transmission in IoT environments by combining OK-EGC with an ASA-DLSTM network. The designed SF-AOA was utilized to efficiently adjust both cryptographic parameters and classification network hyperparameters. This dual optimization approach achieved enhanced classification accuracy and CSI, while reducing encryption/decryption time and memory usage in cryptographic processes. The inclusion of adaptive and sparse attention mechanisms within the ASA-DLSTM improved model efficiency by concentrating on key features, which resulted in better performance for IoT data classification. Ultimately, the integration of optimized cryptography with sophisticated deep learning techniques, directed by SF-AOA, delivered a robust, effective, and scalable solution for secure and dependable data handling in blockchain-enabled IoT systems. The proposed SF-AOA-ASA-DLSTM model achieved 96.5% accuracy at a batch size of 4, which was better than existing models like DNN, SVM, 1DCNN, and SA-DLSTM. Thus, the proposed SF-AOA-ASA-DLSTM model not only enhanced classification accuracy but also ensured efficient computation and scalability, making it highly suitable for real-time, secure data processing in a blockchain-enabled IoT environment.

### Research limitations and future scopes

Though the designed SF-AOA-ASA-DLSTM with OK-EGC and blockchain model attains better performance in secure IoT data transmission and cyberattack identification, some problems remain. The inclusion of deep learning, cryptography, and blockchain poses energy and computational overhead. It may not apply to resource-limited IoT systems like wearable sensors. To resolve this problem, future work will be performed on lightweight developments, including model pruning and employing ASA-DLSTM on edge nodes for minimizing the energy usage and delay. In addition, the current experimental validations are performed in a simulated environment. It doesn’t capture the real-world problems, like unseen attacks. To rectify this, the future work will be performed by including online learning or continual learning mechanisms. It will allow the designed framework to adapt to evolving attack features in real-time. Especially, we will validate the suggested approach with a comprehensive range of IoT datasets and real-world traffic scenarios to assist the generalization ability. Though the developed framework attains efficient performance on benchmark datasets, such as KDD Cup 99 and APA-DDoS, the proposed model may not have the ability to expose the variety and growing nature of sophisticated IoT platforms. So, we will consider traffic patterns from Industrial IoT (IIoT), heterogeneous edge networks, communication protocols, and smart home systems to classify diverse attacks in the future work. In addition, we will extend the proposed framework to manage zero-day attacks, and we will also try to make it adoptable for large-scale deployments by using advanced strategies, which can improve the robustness and practical applicability.

## Data Availability

The data needed for this proposed framework are gathered from two relevant datasets and the descriptions of these datasets are provided below.Dataset 1 (APA-DDoS) is an essential asset for researchers and developers aiming to improve cyber security strategies. The data are collected from the link [https://www.kaggle.com/datasets/yashwanthkumbam/apaddos-dataset](https:/www.kaggle.com/datasets/yashwanthkumbam/apaddos-dataset) accessed on 2025-04-24. This dataset is organized to support the proposed models, providing a wide range of detailed instances of malicious traffic. Such variations are crucial for training machine learning algorithms to identify not only unusual traffic that signals potential attacks but also typical traffic behaviors.Dataset 2(kddcup99.csv) is serves as a prominent standard in the realm of network intrusion detection. The data are collected from the link [https://www.kaggle.com/datasets/venkatakanumuru/kddcup99csv](https:/www.kaggle.com/datasets/venkatakanumuru/kddcup99csv) accessed on 2025-04-24. This dataset is based on simulated network traffic, reflecting different kinds of activity within a military network context. It encompasses not only typical connections but also a range of attack scenarios.

## References

[1] Morarji, C. K. & Sathish Kumar, N. Smart-Grid Monitoring using IoT with Modified Lagranges Key Based Data Transmission, *Intell. Autom. Soft Comput.*, **35**, (2023).

[2] Panahi, U., Cüneyt & Bayılmış Enabling secure data transmission for wireless sensor networks based IoT applications. *Ain Shams Eng. J.*, **14**, 101866, (2023).

[3] Ramyasri, Gudapati, G. R., Murthy, S., Itapu & Mohan Krishna, S. Data transmission using secure hybrid techniques for smart energy metering devices e-Prime-Advances in Electrical Engineering, Electronics and Energy, vol.4, p. 100134, (2023).

[4] Lahane, P. S., Shivaji, R. & Lahane Supply chain management with secured data transmission via improved DNA cryptosystem, Web Intelligence, vol. 22, pp. 401–424, (2024).

[5] Velmurugadass, P., Dhanasekaran, S., Shasi Anand, S. & Vasudevan, V. Quality of Service aware secure data transmission model for Internet of Things assisted wireless sensor networks. *Trans. Emerg. Telecommunications Technol.* **34**, e4664 (2023).

[6] Mondal, S., Ghosh, I. & Das, A. Energy efficient and secure healthcare data transmission in the internet of medical things network. *Microsyst. Technol.* **29**, 539–551 (2024).

[7] Veeramachaneni, V. Fortified Data Transmission for IoT: Leveraging Trustless Networks with Adaptive Security and Energy Efficiency. *J. Adv. Res. Mob. Comput.* **7**, 22–32 (2025).

[8] Bobulski, J., Szymoniak, S. & Pasternak, K. An IoT system for air pollution monitoring with safe data transmission, Sensors, **24**, p. 445, (2024). 10.3390/s24020445 PMC10819453 38257538

[9] Azzedin, F. & Alhazmi, T. Secure data distribution architecture in IoT using MQTT, Applied Sciences, vol. 13, p. 2515, (2023).

[10] Singh, S. & Kumar, D. Energy-efficient secure data fusion scheme for IoT based healthcare system. *Future Generation Comput. Syst.* **143**, 15–29 (2023).

[11] Czeczot, G., Rojek, I. & Mikołajewski, D. Analysis of cyber security aspects of data transmission in large-scale networks based on the LoRaWAN protocol intended for monitoring critical infrastructure sensors, Electronics, **12**, no. 2503, (2023).

[12] Raju, K., Solainayagi, P. & Shathik, J. A. and R. Lalitha A Novel Security Framework for IoT Networks using Blockchain Technology and Adaptive Residual LSTM with Attention Mechanism. *Int. J. Software Eng. Knowl. Eng.*, (2026).

[13] Almalawi, A., Hassan, S., Fahad, A. & Asif Irshad Khan A hybrid cryptographic mechanism for secure data transmission in edge AI networks. *Int. J. Comput. Intell. Syst.* **17**, 24 (2024).

[14] Gou, F. & Wu, J. Novel data transmission technology based on complex IoT system in opportunistic social networks, Peer-to-Peer Networking and Applications, vol. 16, pp. 571–588, (2023).

[15] Michael Raj, T. F., Uma Mageswari, R., Kumar, J. N. & Gowtham Alam, S. A blockchain-based healthcare architecture for secure data management and advanced prediction using an improved wild geese optimized natural extreme gradient boosting algorithm. *Sustainable Computing: Inf. Syst.* **50**, 101301 (June 2026).

[16] Kasetti, S. & Korra, S. Multimedia data transmission with secure routing in M-IOT-based data transmission using deep learning architecture. *J. Comput. Allied Intell.*, pp. 1–13, (2023).

[17] Le Nguyen, B. et al. Privacy preserving blockchain technique to achieve secure and reliable sharing of IoT data, Computers, Materials, & Continua, vol.65, no.87, (2020).

[18] Kaur, M. et al. EGCrypto: A low-complexity elliptic galois cryptography model for secure data transmission in IoT, IEEE Access, vol. 11, pp. 90739–90748, 2023. (2023).

[19] Liu, Y., Wang, X., Zheng, G., Wan, X. & Ning, Z. An AoI-aware data transmission algorithm in blockchain-based intelligent healthcare systems. *Trans. Consumer Electron. IEE Acesses*. **70**, 1180–1190 (2024).

[20] Karim, S. M., Habbal, A., Chaudhry, S. A. & Irshad, A. BSDCE-IoV: Blockchain-based secure data collection and exchange scheme for IoV in 5G environment. *IEEE Access.* **11**, 36158–36175 (2023).

[21] Sutradhar, S., Majumder, S., Bose, R., Mondal, H. & Bhattacharyya, D. A blockchain privacy-conserving framework for secure medical data transmission in the internet of medical things. *Decis. Analytics J.* **10**, 100419 (2024).

[22] Kumar, P. et al. A blockchain-orchestrated deep learning approach for secure data transmission in IoT-enabled healthcare system. *J. Parallel Distrib. Comput.* **172**, 69–83 (2023).

[23] Jiang, S., Cao, J., Wu, H., Chen, K. & Liu, X. Privacy-preserving and efficient data sharing for blockchain-based intelligent transportation systems, Information Sciences, **635**, pp. 72–85, (2023).

[24] Sonkamble, R. et al. Secure data transmission of electronic health records using blockchain technology, Electronics, vol.12, p. 1015, (2023).

[25] Stergiou, C. L., Maria, P., Koidou & Psannis, K. E. IoT-based big data secure transmission and management over cloud system: A healthcare digital twin scenario, Applied Sciences, vol. 13, p. 9165, (2023).

[26] Fu, S., Li, K., Huang, H., Ma, C. & Zhu, Y. Qingsong Fan, and Red-billed blue magpie optimizer: a novel metaheuristic algorithm for 2D/3D UAV path planning and engineering design problems. *Artif. Intell. Rev.* **57**, 6, (2024).

[27] Trojovský, P. & Dehghani, M. Pelican optimization algorithm: A novel nature-inspired algorithm for engineering applications. *Sensors* **22**, 3, (2022). 10.3390/s22030855 PMC8838090 35161600

[28] Falahah, I. et al. Frilled lizard optimization: A novel bio-inspired optimizer for solving engineering applications, Computers, Materials, & Continua, vol.79, no.3631, (2024).

[29] Jebbar, W. A. & Al-Zubaidie, M. Transaction-based blockchain systems security improvement employing micro-segmentation controlled by smart contracts and detection of saddle Goatfish. *SN Comput. Sci.*, **5**, 898, (2024).

[30] Hamadneh, T. et al. Addax Optimization Algorithm: A Novel Nature-Inspired Optimizer for Solving Engineering Applications. *Int. J. Intell. Eng. Syst.*, **17**, 3, (2024).

[31] Tahir, S. Enhancing Identification of IoT Anomalies in Smart Homes Using Secure Blockchain Technology. In Cybersecurity Measures for Logistics Industry Framework, 141–155, IGI Global Scientific Publishing, (2024).

[32] Samanta, D. et al. Cipher block chaining support vector machine for secured decentralized cloud enabled intelligent IoT architecture. *IEEE Access.*, vol.9, pp.98013–98025, (2021).

[33] Qazi, E. U., Haq, A., Almorjan & Zia, T. A one-dimensional convolutional neural network (1D-CNN) based deep learning system for network intrusion detection, Applied Sciences, **12**, 16, (2022).

[34] Aurko Roy, M., Saffar, A. & Vaswani David Grangier Efficient Content-Based Sparse Attention with Routing Transformers, Transactions of the Association for Computational Linguistics, **9**, pp. 53–68, (2021).

[35] Zhang, X., Wang, T. & Yina Guo Qi Xiongand A Dense Long Short-Term Memory Model for Enhancing the Imagery-Based Brain-Computer Interface. *Comput. Intell. Neurosci.*, p. 10 (2021).

[36] Biryukov, A., Christophe De & Cannière Data encryption standard (DES), In Encyclopedia of Cryptography, Security and Privacy, 555–562, Cham: Springer Nature Switzerland, (2025).

[37] Ajagbe, S., Adeola, O. D. & Adeniji Adedayo Amos Olayiwola, and Seun Femi Abiona, Advanced encryption standard (aes)-based text encryption for near field communication (nfc) using huffman compression. *SN Comput. Sci.* **5**, 1, (2024).

[38] Lahraoui, Y., Lazaar, S., Amal, Y. & Nitaj, A. Securing Data Exchange with Elliptic Curve Cryptography: A Novel Hash-Based Method for Message Mapping and Integrity Assurance. Cryptography, 8, no. 2, (2024).

[39] Razzaq, R. H., Al-Zubaidie, M. & Atiyah, R. G. Intermediary decentralized computing and private blockchain mechanisms for privacy preservation in the internet of medical things. *Mesopotamian J. Cybersecur.*, vol.4, pp.152–165, (2024).

